# Proinflammatory isoforms of IL-32 as novel and robust biomarkers for control failure in HIV-infected slow progressors

**DOI:** 10.1038/srep22902

**Published:** 2016-03-15

**Authors:** Mohamed El-Far, Pascale Kouassi, Mohamed Sylla, Yuwei Zhang, Ahmed Fouda, Thomas Fabre, Jean-Philippe Goulet, Julien van Grevenynghe, Terry Lee, Joel Singer, Marianne Harris, Jean-Guy Baril, Benoit Trottier, Petronela Ancuta, Jean-Pierre Routy, Nicole Bernard, Cécile L. Tremblay, Investigators of the Canadian HIV Slow Progressor Cohort, Jonathan Angel, Brian Conway, Pierre Côté, John Gill, Lynn Johnston, Colin Kovacs, Mona Loutfy, Kenneth Logue, Alain Piché, Anita Rachlis, Danielle Rouleau, Bill Thompson, Réjean Thomas, Sylvie Trottier, Sharon Walmsley, Wendy Wobeser

**Affiliations:** 1 CHUM-Research Centre, Montréal, QC, Canada; 2 Département de Microbiologie, Infectiologie et Immunologie, Faculté de Médecine, Université de, Montréal, QC, Canada; 3 Caprion, Montréal, QC, Canada; 4 INRS-Institut Armand Frappier, Laval, QC, Canada; 5 CIHR Canadian HIV Trials Network, St. Paul’s Hospital, Vancouver, BC, Canada; 6 AIDS Research Program, St. Paul’s Hospital, Vancouver, BC, Canada; 7 Clinique Médicale Quartier Latin, Montréal, QC, Canada; 8 Clinique Médicale l’Actuel, Montréal, QC, Canada; 9 Division of Hematology and Chronic Viral Illness Service, McGill University Health Centre, Montréal, QC, Canada; 10 Research Institute, McGill University Health Centre, Montréal, QC, Canada; 11 Ottawa Health Research Institute, Ottawa Hospital, ON, Canada; 12 Downtown Infectious Diseases Clinic, Vancouver, BC, Canada; 13 Clinique médicale du Quartier Latin, Montréal, QC, Canada; 14 Southern Alberta Clinic, Calgary, AB, Canada; 15 Capital District Health Authority, Halifax, NS, Canada; 16 Maple Leaf Medical Clinic, Toronto, ON, Canada; 17 St-Clair Medical Associates, Toronto, ON, Canada; 18 Département de microbiologie et d’infectiologie, Centre hospitalier de l’Université de Sherbrooke (CHUS), QC, Canada; 19 SunnyBrook Health Sciences Centre, Toronto, ON, Canada; 20 Centre hospitalier de l’Université de Montréal, Notre-Dame, QC, Canada; 21 Lawson Research Inc, St. Joseph’s Health Care, London, ON, Canada; 22 Clinique médicale L’Actuel, Montréal, QC, Canada; 23 Centre de recherche en infectiologie (CRI), Centre hospitalier de l’Université Laval, Québec, Canada; 24 Toronto General Hospital & Research Institute, ON, Canada; 25 Queen’s University, Kingston, ON, Canada

## Abstract

HIV-infected slow progressors (SP) represent a heterogeneous group of subjects who spontaneously control HIV infection without treatment for several years while showing moderate signs of disease progression. Under conditions that remain poorly understood, a subgroup of these subjects experience failure of spontaneous immunological and virological control. Here we determined the frequency of SP subjects who showed loss of HIV control within our Canadian Cohort of HIV^+^ Slow Progressors and identified the proinflammatory cytokine IL-32 as a robust biomarker for control failure. Plasmatic levels of the proinflammatory isoforms of IL-32 (mainly β and γ) at earlier clinic visits positively correlated with the decline of CD4 T-cell counts, increased viral load, lower CD4/CD8 ratio and levels of inflammatory markers (sCD14 and IL-6) at later clinic visits. We present here a proof-of-concept for the use of IL-32 as a predictive biomarker for disease progression in SP subjects and identify IL-32 as a potential therapeutic target.

Infection with the human immunodeficiency virus (HIV) remains a global health challenge despite the remarkable success of combined antiretroviral therapy (cART) to significantly reduce both mortality and morbidity in the infected population. However, even with near-complete viral suppression by the current classes of treatment, curing HIV infection remains unachievable and patients must adhere to lifelong treatment. This is largely due to the persistence of replication-competent HIV in latent viral reservoirs that are resistant to the current regimens, and to the capacity of these reservoirs to reinitiate infection upon cessation of therapy^1^,^2^,^3^. Both long-term exposure to treatment and persistence of viral infection are likely to have a clinical cost as evidenced by the treatment-associated toxicities, persistent inflammation, immune dysfunction, cardiovascular and neurologic disorders and pre-mature aging seen in treated subjects^4^,^5^. Furthermore, the failure of different vaccine trials aiming to prevent HIV infection^6^ and the partial success of others^7^ together highlight the critical need for novel and unconventional therapies. For these reasons, there is a renewed interest in novel immunological strategies that aim to eliminate viral reservoirs and to strengthen immune responses able to control viral replication after infection, thereby limiting ART exposure and achieving a functional cure^8^,^9^.

Natural and sustained immunological responses are indeed observed in a subset of HIV-infected individuals who spontaneously control HIV infection without ART for several years while showing moderate signs of disease progression. These subjects represent the HIV-infected slow progressors (SP), including the rare Elite Controller (EC) subgroup, which constitutes less than 1% of the HIV-infected population^10^,^11^. The low rate of transmission and slow disease progression associated with lower levels of HIV-RNA and prolonged high CD4^+^ T-cell counts make the study of these SP subjects of particular interest to inform and fuel potential strategies that support a functional cure for HIV infection^12^,^13^,^14^. Genome-wide association studies have implicated the major histocompatibility complex (MHC) class I region in natural control of HIV viral load (VL)^15^. A higher frequency of HIV-infected subjects carrying the MHC class I alleles such as HLA-B^*^27 and HLA-B*57 was observed in SPs compared to typical progressors (TP). Cytotoxic CD8^+^ T-cells that recognize complexes of these protective MHC class I antigens and HIV epitopes are particularly effective at controlling HIV replication^16^,^17^,^18^,^19^. However, many SP subjects do not carry protective MHC class I alleles^20^. Furthermore, some SPs, including those carrying protective MHC class I alleles, fail to maintain long-term control and eventually exhibit HIV disease progression^21^,^22^. This suggests that other immunological and virological parameters are also involved in the remarkable capacity of these SP subjects to control HIV infection and that these parameters may not be sustained forever. Examining host and viral parameters in SPs before and after loss of control, provides an opportunity to identify the mechanisms underlying enhanced immunological and virological control and its loss in these SPs who begin to progress. With this in mind, the Canadian Cohort of HIV^+^ Slow Progressors (CCHSP) was established in Canada to better characterize correlates of HIV control among both aviremic and viremic SPs.

In the current study we investigated the rate of CD4^+^ T-cell decline in subgroups of the CCHSP, which differed from each other in the level of virological control, and identified subjects who experienced loss of virological control accompanied by significant declines in CD4^+^ T-cell counts. We employed genome-wide transcriptional analysis on peripheral blood from these later subjects, before and after loss of control, to identify and validate biomarkers and predictive factors associated with disease progression in HIV-infected SP.

## Results

### Rate of CD4 decline in HIV-infected SPs

HIV-infected SPs included Elite controllers (EC, VL ≤ 50 HIV RNA copies/ml of plasma and CD4 counts > 500 cells/mm^3^ at study entry), Virologic Controllers, (VC, VL 51–3,000 copies/ml of plasma and CD4 counts > 500 cells/mm^3^ at study entry) and Non-virologic Controllers (NVC, VL > 3,000 copies/ml of plasma and CD4 counts > 500 cells/mm^3^, for >7 years) (Table 1). Using the definitions of HIV control as described above, there were n = 45 EC, n = 68 VC and n = 33 NVC in our cohort (demographic and disease history characteristics of these subgroups are presented in Supplementary Table S1). A control group of Typical Progressor (TP) HIV-infected subjects (n = 490 subjects) was used for comparisons. These subjects were enrolled in the Montreal Primary Infection (PI) or PRIMO cohort, which enrolls individuals during the first year of infection and follows them for up to 2 years (Supplementary Table S1).

**Figure 1:**
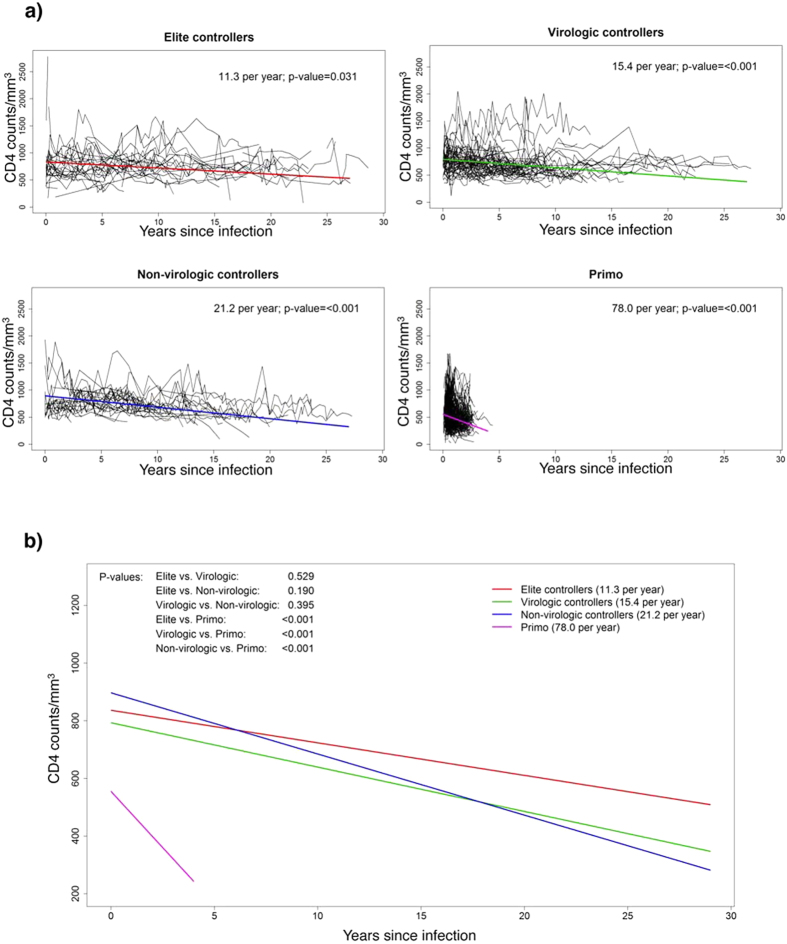
Decline of CD4^+^ T-cell counts in the different slow progressor (SP) subgroups. (**a**) Mixed effects regression analysis on CD4 decline for Elite controllers (n = 45) (Left upper panel), Virologic controllers (n = 68) (Right upper panel), Non-virologic controllers (n = 33) (Left lower panel) compared to Typical progressors (Primo) (n = 490) (Right lower panel). Black lines are the individual CD4 profiles and colored lines are the estimated CD4 decline from the mixed effects regression analysis. (**b**) Slope of CD4 decline from the same 4 groups of subjects as in A.

By using mixed effects regression analysis on historical and prospective data, we observed that the estimated rate of CD4 decline varies according to the SP study subgroups defined by VL. EC, VC and NVC subjects exhibited annual rates of CD4 decline of 11.3, 15.4 and 21.2 cells/mm^3^. The rate of CD4 count change for each of these subgroups was significantly less than “zero” for each of these subject groups (p = 0.031, p < 0.001 and p < 0.001 for EC, VC and NVC, respectively) (Fig. 1a). However, the rates of CD4 decline between EC, VC and NVC were not significantly different (Fig. 1b). The TP group had the highest rate of CD4 decline with an annual loss of 78 cells/mm^3^ (p < 0.001) and represented a faster decline compared to that of each of the SP subgroups (Fig. 1b). We also evaluated the effect of a variety of baseline covariates (gender (Supplementary Fig. S1a), age at diagnosis (Supplementary Fig. S1b), race (Supplementary Fig. S2) and modes of transmission (Supplementary Fig. S3)) on the CD4 slope. None of these covariates were found to have a significant impact on the CD4 slope (p-values were >0.2 for all the covariates in the univariate regression analysis and thus covariates were not included in the main analysis.

At the time of analysis, HLA typing data were available for 42/45 of EC, 63/68 of VC and 30/33 of NVC. The frequency of subjects possessing protective alleles within each SP subgroup is presented in Table 2. Within the SP cohort, the presence of HLA-B^*^27 protective allele was found to differ significantly between SP subgroups with the EC subjects having the highest prevalence (28.6%) compared to 17.5% and 3.3% for VC and NVC, respectively (Fisher’s exact test p = 0.022). Of interest, 17.8% (24/135) of all SP subjects carried the protective HLA-B*27 allele compared to 8.1% (37/455) of TP (p = 0.001, Fisher’s exact test). HLA-B*57 was carried by 22.2% (30/135) of SP compared to 5.5% (25/455) of TPs (p < 0.001, Fisher’s exact test).

### Identification of SP subjects who experience failure of immunological and virological control

**Figure 2:**
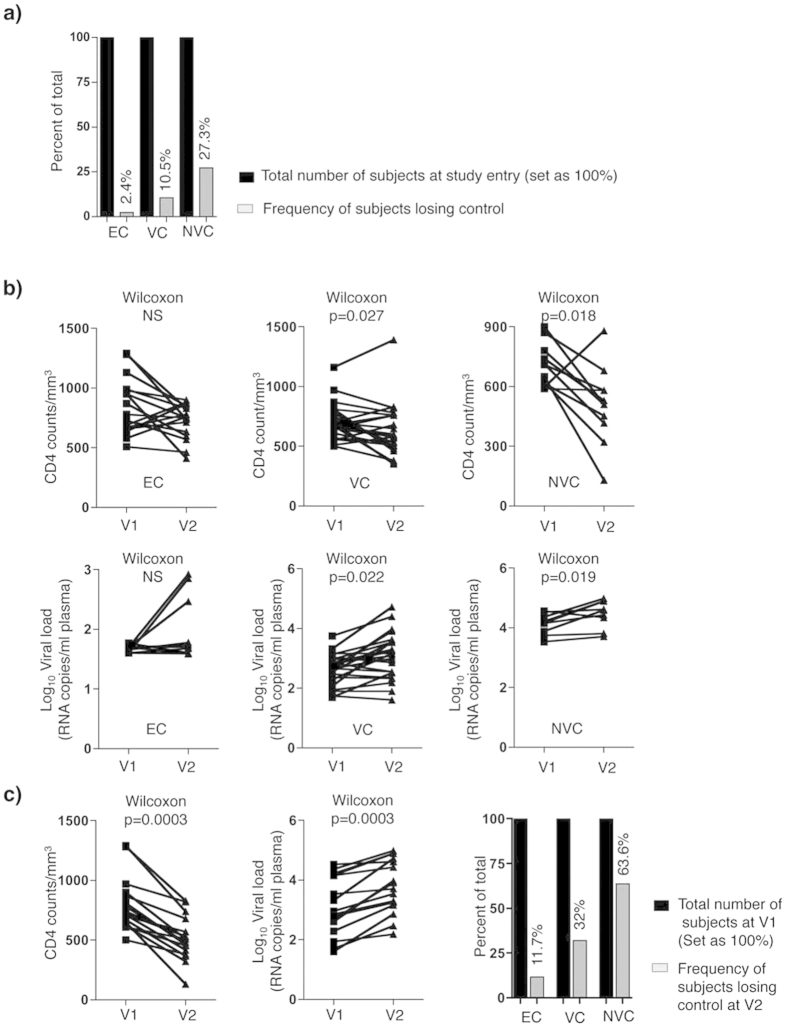
Longitudinal determination of CD4^+^ T-cell counts and viral load (VL) of HIV-infected SP subgroups. (**a**) Frequency of EC, VC and NVC subjects with statistically significant negative slopes for CD4 decline and increased VL calculated on the total number of visits since infection (shown in Table 3). (**b**) CD4 counts (Upper panels) and Log_10_ VL (Lower panels) in EC (n = 17), VC (n = 25) and NVC (n = 11) at two clinic visits, V1 (all subjects having CD4 counts ≥ 500 CD4^+^ T-cells/mm^3^ and VL that corresponds to criteria used to classify each of the subgroups) and V2 (an average time interval from V1 of 30 ± 16, 34 ± 25 and 33 ± 15 months for EC, VC and NVC, respectively). (**c**) CD4 counts (Left panel) and Log_10_ VL (Middle panel) at V1 and V2 for SP subjects who lost HIV control (CD4 count decrease of ≥100 cells/mm^3^ combined with any increase in Log_10_ VL). Right panel: Frequencies of EC, VC and NVC subjects losing control between V1 and V2 (EC n = 2/17, VC n = 8/25, and NVC n = 7/11). *P* values were calculated by non-parametric paired two-tail test (Wilcoxon) in B and C. EC = Elite Controller; VC = Virologic Controllers; NVC = Non Virologic Controllers.

We determined the frequency of SP subjects who experienced HIV disease progression by monitoring longitudinal CD4 count and VL changes. We defined the failure of control with a primary criterion of a negative slope of CD4 count (significantly different from “zero”) combined with a significant increase in VL based on historical and prospective clinical records from the time of infection to time of the last recorded clinic visit for all subjects within the SP cohort (subjects having <3 clinic visits were excluded from the analysis, n = 4). As shown in Table 3 and Fig. 2a, only 17 subjects (EC (n = 1/42, 2.4%), VC (n = 7/67, 10.5%) and NVC (n = 9/33, 27.3%)) had a significant (p < 0.05) negative CD4 count slope combined with a significant (p < 0.05) positive slope for VL. These statistics were determined on a combination of historical (since the time of infection and prior to study entry) and actual clinical data (following enrolment to the study) from the SP subgroups. However, to identify clinical visits, after enrolment to the study, from which biological samples were available and could be used for further experimental investigation on failure of control, both CD4 counts and VL were measured from two longitudinal clinic visits on n = 53 subjects including a representative number from each subgroup, EC (n = 17), VC (n = 25) and NVC (n = 11). Visit 1 (V1) corresponded to a time point close to study entry at which CD4 counts were ≥500 CD4^+^ T-cells/mm^3^ and VL within the range used to categorize subjects as EC, VC or NVC. Visit 2 (V2) corresponded to an average ± standard deviation time interval of 30 ± 16, 34 ± 25 and 33 ± 15 months for EC, VC and NVC, respectively. CD4 counts for EC, VC and NVC at V1 were 848 ± 234, 705 ± 144 and 708 ± 107 cells/mm^3^, respectively, and average Log_10_ VLs were 1.66 ± 0.05, 2.65 ± 0.51 and 4.17 ± 0.32, respectively. At V2, CD4 counts for EC, VC and NVC were 725 ± 144, 622 ± 211 and 505 ± 192 cells/mm^3^, respectively, and average Log_10_ VLs were 1.8 ± 0.44, 3.1 ± 0.76 and 4.48 ± 0.43, respectively. A decline in CD4 counts and an increase in VL was observed for EC, VC and NVC but was significant for only the VC and NVC subgroups (p = 0.027 and 0.018, respectively for CD4 count, Fig. 2b, Upper panels, and p = 0.022 and p = 0.019, respectively for VLs, Fig. 2b, Lower panels). By fixing a threshold for the loss of control between the two visits as a decline in CD4 counts ≥100 cells/mm^3^ combined with any increase in the Log_10_ VL, only 2 of 17 (11.7%) EC subjects, 8 of 25 (32%) VC, and 7 of 11 (63.6%) of NVC for a total of 17 of 53 (32%) SP subjects met the criteria for disease progression (Fig. 2c). Six out of these 17 subjects were also identified as having a CD4 count decline with a VL increase and thus this was evidence of HIV disease progression based on their historical and prospective data (Table 3) and Fig. 2a.

At the time of analysis, HLA typing was available for 15 out of the 17 SP subjects losing control between V1 and V2. Of these, 7 subjects (46%) had at least one of the protective HLA class I alleles, HLA-B^*^27, HLA-B*51 or HLA-B*57 (Supplementary Table S2).

Together these data showed that the EC subjects are more resistant to disease progression than VC and NVC subjects. These results also revealed that that loss of control occurs in SP subjects despite carrying protective HLA alleles.

### Identification of a molecular signature associated with failure of control in SP subjects

**Figure 3:**
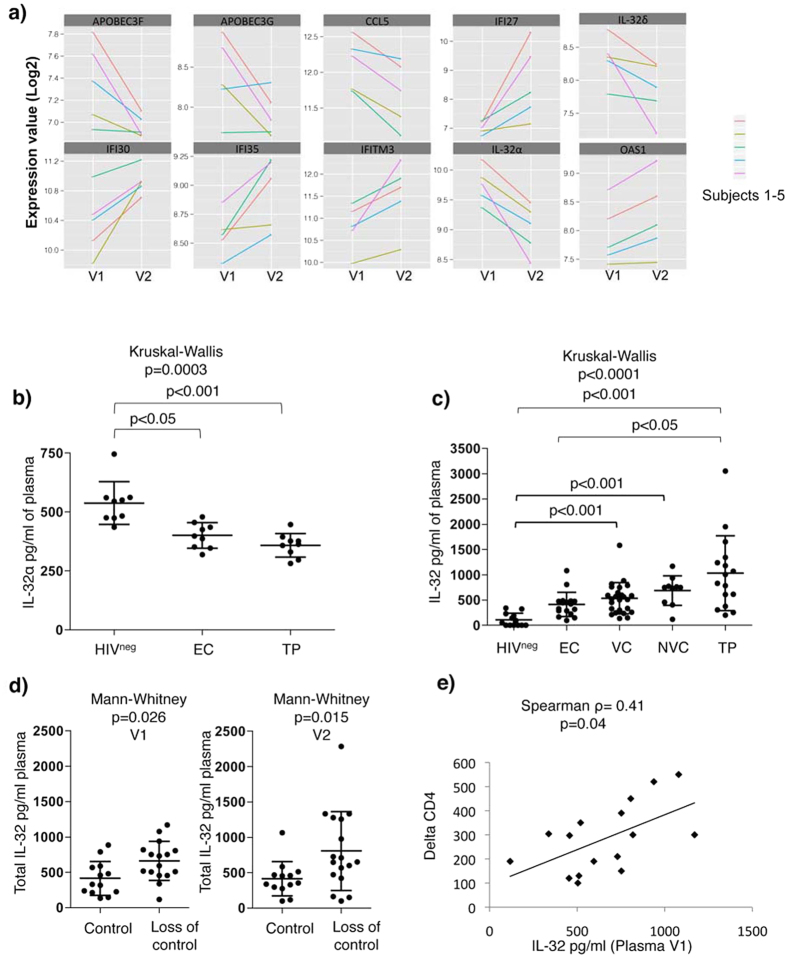
Loss of HIV control in SP subjects is associated with decreased IL-32α/δ and increased non-α non-δ IL-32 isoforms. (**a**) Line plot showing a summary of Log_2_ fold change in gene expression of 10 selected genes including IL-32α and δ from microarray analysis on n = 5 SP subjects who lost control (VC, n = 2 and NVC, n = 3). (**b**) Plasma levels of IL-32α measured by ELISA on EC and TP subjects compared to HIV^neg^ controls (n = 9/group). (**c**) Total IL-32 (α, β, γ and δ) measured by ELISA on HIV^neg^ (n = 13), EC (n = 17), VC (n = 25), NVC (n = 11) compared to TP subjects (n = 16). (**d**) Total IL-32 in plasma from subjects losing control (n = 17) compared to subjects showing no decrease in CD4 counts or increase in VL (n = 13) at both V1, before loss of control (Left Panel) and V2, after loss of control (Right panel). The lines and error bars through each data set represent the mean ± SD for the group. (**e**) Correlation between the total levels of plasma IL-32 at V1 and the change in CD4 counts at V2 (delta CD4) for the n = 17 SPs who lost HIV control. Kruskal-Wallis and Dunn’s post tests were used to assess the significance of between-group differences in panels (**b**,**c**) Mann-Whitney tests were used assess the significance of between-group differences in panel (**d**) Spearman correlation tests were used to assess the significance correlations between the 2 parameters tested in panel (**e**).

To gain further insight into the potential mechanisms that govern the loss of immunological (CD4 counts) and HIV control (viral load), we identified 5 subjects (VC, n = 2 and NVC, n = 3) who experienced combined loss of CD4 counts (average loss of 211 CD4^+^ T-cells/mm^3^) and increased VL (average increase of 20 fold) between V1 and V2 (visits following study entry selected as described in the previous section). Peripheral blood mononuclear cells (PBMCs) collected from these subjects at V1 and V2, before and after loss of control, respectively, were used for genome-wide transcriptional profiling using the Illumina microarray technology. Our analysis identified 1,381 probe sets corresponding to 1,268 genes that were differentially expressed between V1 and V2 (p-value < 0.05). By applying a cut-off fold change (FC) of 1.3, we identified 207 genes down-regulated and 83 genes up-regulated at V2 compared to V1 among the differentially expressed genes. Of note, among the down-regulated genes, several members of the innate antiviral responses were identified such as APOBEC3G (FC = −1.4, p = 0.03), APOBEC3F (FC = −1.32, p = 0.012), CCL5 (FC = −1.34, p = 0.001) and IL-32 (alpha and delta isoforms) (FC = −1.7, p = 0.0007 and FC = −1.4, p = 0.022, respectively) (Fig. 3a). Other transcripts previously linked to T-cell activation such as CD160, ITK and the IL-7 receptor (IL-7R) also showed a marked decrease (Supplementary Fig. S4).

Of these modulated genes, down-regulation of IL-32α and δ isoforms at V2 was of particular interest as IL-32 was previously shown to play an intracellular antiviral role, mainly mediated by interferon-induced genes (ISGs)^23^,^24^. Consistently, we also observed a significant increase in several ISGs such as IFI27, IFI30, IFI35, IFITM3 and OSA1 (Fig. 3a), suggesting an ongoing interferon response, likely due to the increased HIV replication. Since the IL-32α and δ isoforms are down-modulated between V1 and V2 this suggests that they are not likely involved in mediating expression of the interferon-induced signalling linked to HIV infection.

In accordance with the decrease of IL-32α mRNA in our transcriptomic analysis, the ELISA quantification of IL-32α soluble protein in plasma from EC and TP subjects compared to HIV-uninfected controls (HIV^neg^) showed significantly lower IL-32α levels (p < 0.05 and p < 0.001, respectively). There was a tendency for higher IL-32α levels in ECs compared to that in TPs, although the difference did not achieve statistical significance (Fig. 3b). Of note, we were not able to measure the IL-32δ at the protein level due to the lack of specific antibodies. Plasma IL-32 was further measured using a set of antibodies having the capacity to recognize the 4 prototypic isoforms, α, β, γ and δ (that we refer to here as total IL-32). Intriguingly, the total IL-32 levels were significantly higher in TPs compared to both HIV^neg^ and EC subjects (p < 0.001 and p < 0.05, respectively, Fig. 3c). This was also the case for total IL-32 levels quantified in cell lysate of PBMCs collected from EC, TP and HIV^neg^ subjects (Supplementary Fig. S5a). Furthermore there was a positive correlation between cell-associated and plasma levels of total IL-32 measured from the same subjects (Supplementary Fig. S5b). These results suggest that the IL-32 isoforms other than IL-32α and δ, most likely the IL-32 β and γ isoforms contribute to the pool of total IL-32 in plasma and cells from HIV infected subjects. Of note, the isoforms IL-32β and γ in contrast to IL-32α and δ exhibit pro-inflammatory features^25^ thus raising the possibility that their over-expression is likely linked to the loss of control. Interestingly, plasma levels of total IL-32 were lower in EC subjects compared to TP (p < 0.05, Fig. 3c). These results suggest a positive association between the levels of the IL-32 isoforms, other than IL-32α and δ, and VL. This was further confirmed by measuring the level of total plasma IL-32 in SP subjects losing control (subjects identified in Fig. 2c, n = 17). As expected, total IL-32 was significantly higher in SP subjects losing virological control compared to subjects from the same cohort who maintained unchanged VL and a stable CD4 between V1 and V2 (Fig. 3d). It was notable that total IL-32 levels at V1 were significantly higher in the SPs who were going to lose HIV control at V2 than they were in SP who maintained VL control (Fig. 3d) and these total IL-32 levels were positively correlated with the CD4 count change from V1 to V2 (delta CD4) (Spearman ρ = 0.41, p = 0.04, Fig. 3e).

Together, these results showed that while loss of CD4 counts and increased viremia in a subgroup of SP subjects is associated with a decrease in the IL-32α (confirmed at the protein level) and IL-32δ (data from the transcriptomic analysis), there was a significant increase in the non-α/non-δ IL-32 isoforms that high likely included the pro-inflammatory isoforms β and γ. Furthermore, there was a significant correlation between levels of total IL-32 and the degree of CD4 count decline as measured by magnitude of the decrease in CD4 counts from V1 to V2.

### HIV infection increases IL-32 production

**Figure 4:**
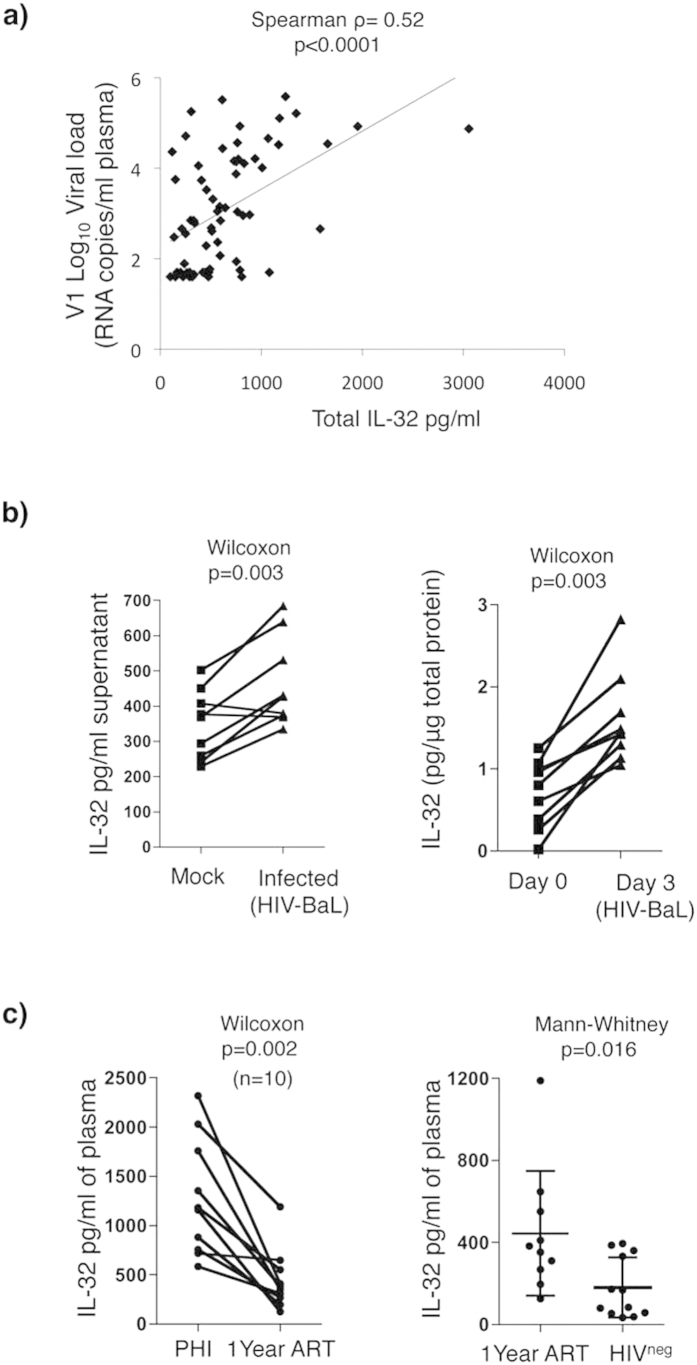
HIV infection induces expression of IL-32 in human PBMCs. (**a**) Correlation between total IL-32 and VLs from HIV-infected SP (n = 53) (EC, VC, NVC) and TP subjects (n = 16) (subjects shown in Fig. 3c). Spearman correlation test was used to assess the significance correlations between IL-32 and HIV VL. (**b**) Human PBMCs from n = 9 HIV-uninfected donors were either stimulated with PHA (0.25μg/ml) and IL-2 (100 units/ml) and infected with HIV-BaL (Left panel) or resting cells were infected without stimulation (Right panel). Total IL-32 was measured in the supernatant of activated cells (Left panel) or from cell lysate of non-stimulated cells (Right panel). (**c**) Total plasma IL-32 was measured in n = 10 subjects within 3 mos of HIV infection and after 1 year of ART treatment (Left panel). Total plasma IL-32 was measured in the same 10 subjects treated with ART for 1 yr and in 12 HIV^neg^ donors (n = 12) (Right panel). The significance of between-group differences was assessed using a Wilcoxon test in panel (**b**) and the Left panel of (**c**). A Mann-Whitney test was used to assess the significance of between-group differences in panel (**c**) (Right panel).

Total IL-32 levels in the different subgroups of HIV-infected subjects (EC, VC, NVC and TP Fig. 3c) were highly and significantly correlated with HIV VLs from these subjects (Spearman ρ = 0.52, p < 0.0001, Fig. 4a). These results suggest that enhanced expression of IL-32 in the periphery of HIV-infected subjects is likely driven directly by viral infection. To test this hypothesis, total PBMCs from HIV^neg^ donors were stimulated with PHA and IL-2 for 48 h followed by infection with the laboratory strain HIV-BaL for 3 extra days. In another set of experiments, total PBMCs were directly infected for 3 days without previous stimulations. As shown in Fig. 4b infection of either stimulated or unstimulated cells led to a significant up-regulation total IL-32 measured in the supernatant or as cell-associated protein, respectively (p = 0.003 for both). We further measured the levels of IL-32 in subjects with recent HIV infection. Total IL-32 levels in plasma from recently infected viremic subjects (n = 10 subjects infected for ≤3 months) were higher than that in the same subjects treated for 1 year with ART (p = 0.002) (Fig. 4c, Left panel), thus emphasizing the link between IL-32 and viral replication. Results in Fig. 4c (Right panel) demonstrate that even following 1 year of treatment, the total IL-32 levels remained significantly higher compared to HIV^neg^ donors (n = 10 PHI and n = 12 HIV^neg^ donors, p = 0.016). Together, our observations suggest that IL-32 is induced early after HIV infection and is not normalized by viral suppressive ART.

### Plasma levels of total IL-32 predict inflammation, CD4 decline and increased VL in SP subjects

**Figure 5:**
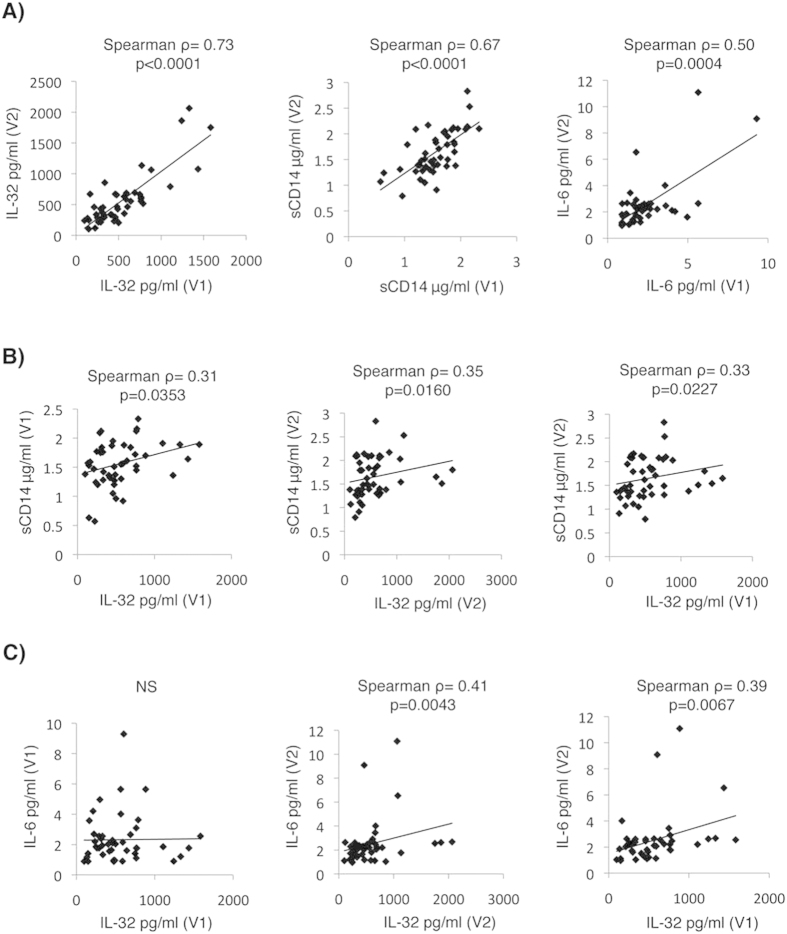
Persistent levels of IL-32 predict inflammatory and clinical markers. (**A**) Correlations between individual inflammatory markers, total levels of IL-32 (Left panel), sCD14 (Middle panel) and IL-6 (Right panel), measured in plasma from the same EC (n = 19), VC (n = 22), NVC (n = 6) subjects at V1 and V2. Correlations between total IL-32 at V1 and V2 with sCD14 (**B**) and IL-6 (**C**) at V1 and V2 on the same subjects as in panel (**A)**. A Spearman correlation test was used to assess the significance of correlations between the 2 measured parameters. The correlation coefficient (ρ) and p-value for.

Our observation that total IL-32 levels are high in early HIV infection (Fig. 4c) and in SP subjects who experience loss of HIV control (Fig. 3d) and that these levels positively correlate with the decrease in CD4^+^ T-cell count from V1 to V2 (Fig. 3e), together with the proinflammatory nature of IL-32 non-α/non-δ isoforms^25^, led us to hypothesize that IL-32 levels may predict the loss of control in the general population of SP subjects. To test this hypothesis, we first confirmed the chronicity of IL-32 secretion by measuring its levels longitudinally in plasma from the SP subjects. Subjects with confirmed loss of control between V1 and V2 (Fig. 2c) were excluded from this analysis. We also measured other inflammatory markers such as sCD14, a marker of innate immune activation and a predictor of disease progression and mortality in ART-treated subjects^26^, and IL-6, an inflammatory marker that predicts ongoing HIV replication *in vivo*^27^. As shown in Fig. 5A, levels of total IL-32 at V1 and V2 were positively correlated in the general population of SPs (Left panel, Spearman ρ = 0.73, p < 0.0001). Similarly, levels of both sCD14 and IL-6 at V1 and V2 were positively correlated (Middle and Right panels, respectively, Spearman ρ = 0.67, p < 0.0001 for sCD14, and Spearman ρ = 0.50, p = 0.0004 for IL-6). Moreover, total IL-32 levels at both V1 and V2 were positively and significantly correlated with sCD14 levels in plasma from the same time points (Fig. 5B, Left and Middle panels). Most importantly, IL-32 levels at V1 significantly correlated with both sCD14 and IL-6 levels in plasma from V2 (Spearman ρ = 0.33, ρ = 0.39, and p = 0.0227 and p = 0.0067, respectively) (Fig. 5B,C, Right panels). These results suggest that IL-32 levels at V1 can predict levels of inflammatory markers at later time points.

**Figure 6:**
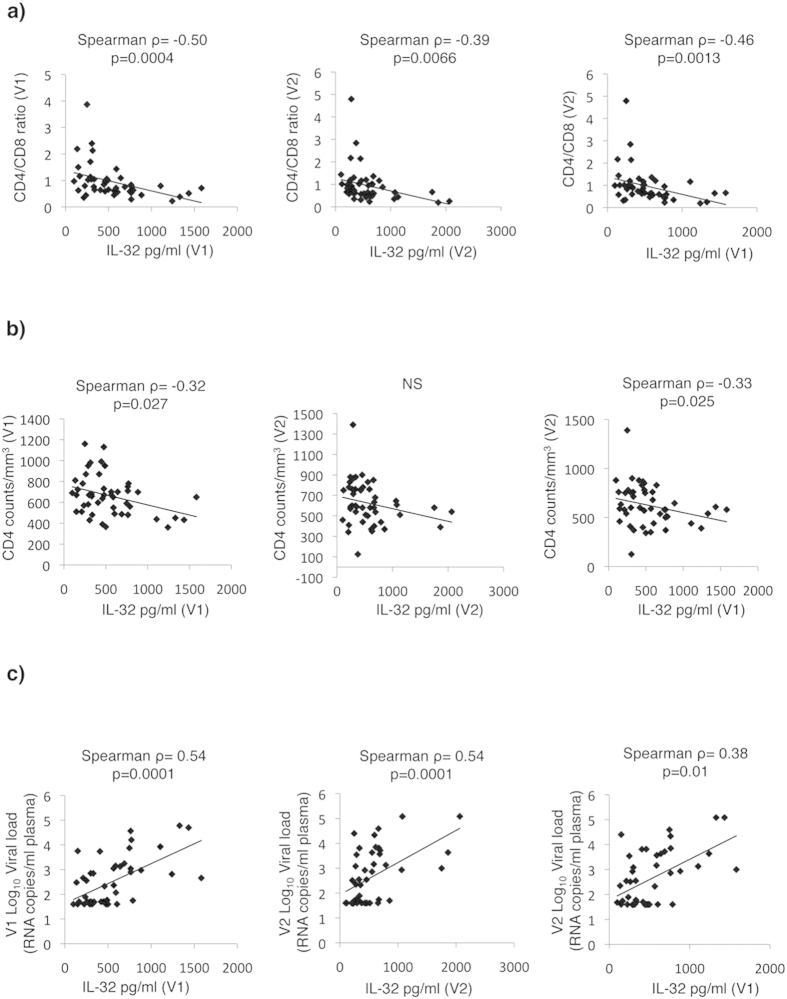
Plasma IL-32 levels predict typical clinical parameters associated with disease progression in SP subjects. Correlations between plasma levels of total IL-32 measured for the same subjects as in Fig. 5 and CD4/CD8 ratio (**a**), absolute CD4^+^ T-cell counts (**b**) and HIV Log_10_ VL (**c**) at V1 (Left panels), V2 (Middle panels) and V1 *versus* V2 (Right panels). A Spearman correlation test was used to assess the significance of correlations between the 2 measured parameters. The correlation coefficient (ρ) and p-value for each comparison are shown over the graphs.

In HIV infection the CD4/CD8 ratio is usually inverted (<1) and is often used as a marker for HIV-associated immune dysfunction^28^,^29^,^30^. This ratio was negatively and significantly correlated with IL-32 levels at both V1 and V2 (Spearman ρ = −0.50, ρ = −0.39, and p = 0.0004 and p = 0.0066, respectively, Fig. 6a Left and Middle panels). Furthermore, IL-32 levels at V1 were negatively correlated with CD4/CD8 at V2 (Spearman ρ = −0.46, and p = 0.0013, Fig. 6a, Right panel). IL-32 levels at V1 further correlated negatively and significantly with CD4 counts at V1 (Spearman ρ = −0.32, and p = 0.027, Fig. 6b, Left panel). At V2, IL-32 showed a non-significant trend towards being negatively correlated with CD4 counts. However, as observed for the sCD14, IL-6 and CD4/CD8 ratio, IL-32 at visit 1 negatively and significantly predicted CD4 counts at visit 2 (Spearman ρ = −0.33, and p = 0.025, Fig. 6b, Right panel). Interestingly, neither sCD14 nor IL-6 at visit 1 could significantly predict CD4 or CD4/CD8 ratio at visit 2 (Supplementary Figs S6 and S7).

Based on our observation that HIV-infected typical progressors (viremic) have higher IL-32 levels compared to HIV-infected aviremics (EC) (as shown in Fig. 3c) and the significant correlation between IL-32 and VL (Fig. 4a), we expected that IL-32 levels at V1 to positively predict higher HIV VLs at V2. As shown in Fig. 6c (Left and Middle panels) total IL-32 levels positively correlated with HIV Log_10_ VL at both V1 and V2 (Spearman ρ = 0.54, and p < 0.0001 for both). IL-32 at V1 further predicted the HIV burden at visit 2 (Spearman ρ = 0.38 and p = 0.01 (Fig. 6c, Right panel). Finally, no correlation was observed based on age, sex or time between V1 and V2 (Supplementary Fig. S8).

Collectively, our results show that total IL-32 is a robust biomarker for loss of control, increased HIV burden and inflammation in the SP cohort.

### IL-32γ compared to IL-32α has a proinflammatory profile consistent with enhanced HIV replication

**Figure 7:**
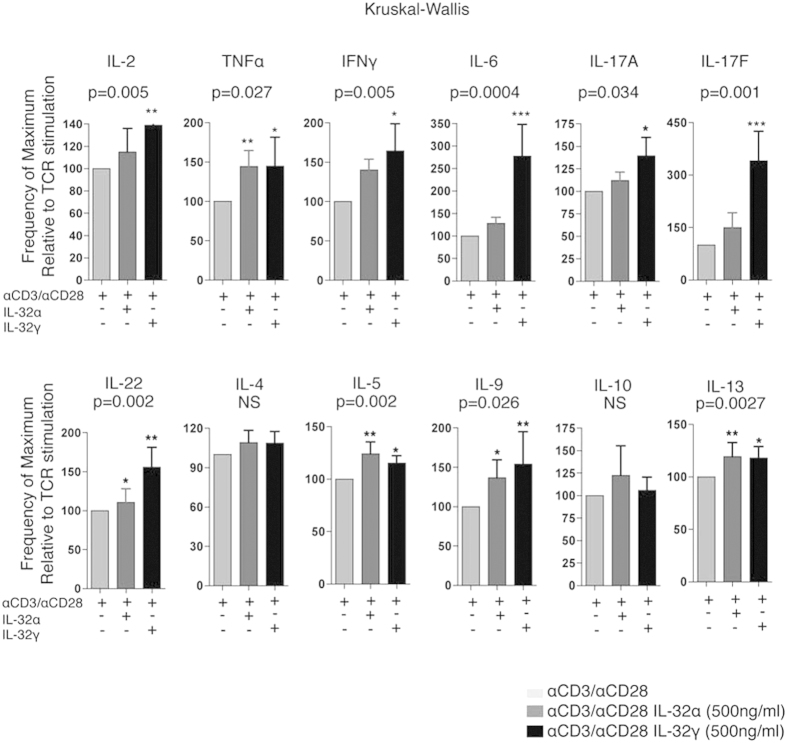
IL-32γ enhances Th1 and Th17 cytokine secretion from CD4^+^ T-cells stimulated through the T-cell receptor. CD4^+^ T-cells were isolated from HIV^neg^ (n = 3) and HIV-infected ART-treated subjects (n = 3) by negative selection and stimulated with plate-bound anti-CD3 (0.5 μg/ml) and anti-CD28 (0.5 μg/ml) in the presence or absence of either IL-32α (500 ng/ml) or IL-32γ (500 ng/ml). Shown on these bar graphs are cytokine levels secreted following activation through the TCR alone (set at 100%) or in the presence of either IL-32α or IL-32γ. Bar height and error bars represent the mean ± SD for a combined group of n = 3 HIV^neg^ and n = 3 HIV-infected ART-treated subjects. Kruskal-Wallis and Dunn’s post tests were used to assess the significance of between group cytokine levels when IL-32α or IL-32γ were present in addition to the anti-CD3/CD28 TCR stimulus. *p < 0.05, **p < 0.001, ***p < 0.0001.

IL-32 was initially described as an intracellular anti-viral factor^23^. However, our observations on correlations between total IL-32 (essentially non-α/non-δ isoforms) and inflammatory markers suggest that these isoforms may be implicated in mechanisms that promote HIV replication instead of exerting intracellular antiviral effects. This hypothesis is supported by earlier observations showing that IL-32γ-mediated inflammation can induce immune suppression by activating the production of Indoleamine 2,3-dioxygenase, IDO1^31^. We next investigated the impact of IL-32γ, compared to IL-32α (the only available recombinant isoforms of IL-32) on the activation of primary T-cells by measuring a panel of secreted inflammatory and anti-inflammatory cytokines following TCR activation. The panel included IL-2, IL-4, IL-5, IL-6, IL-9, IL-10, IL-13, IL-17 A, IL-17 F, IL-22, TNFα and IFNγ). CD4^+^ T-cells isolated from HIV^neg^ donors or HIV-infected subjects were stimulated with CD3/CD28 antibodies to engage the T-cell receptor in the presence or absence of either IL-32α or IL-32γ. Supernatants collected 48 h after stimulation showed that both IL-32α and IL-32γ had an additive effect on TCR stimulation. The effect of IL-32γ on the cytokine production by T-cells from both HIV^neg^ and HIV-infected subjects was superior to that of IL-32α (Fig. 7 and Supplementary Table S3). In line with previous reports on other cell types^32^,^33^, IL-32γ induced significantly higher levels of IL-6 in CD4^+^ T-cells, consistent with the *in vivo* data depicted in Fig. 5C. More importantly, IL-32γ induced higher levels of IL-17 A and IL-17 F upon stimulation of CD4^+^ T-cells, which indicates a specific activation of Th17 cells, a CD4^+^ T-cell subset documented to be highly permissive to HIV infection^34^,^35^,^36^. Similar results were obtained from CD4^+^ T-cells isolated from both HIV^neg^ and HIV^+^ subjects (Supplementary Table S3).

Together, these results demonstrated the greater potency of IL-32γ compared to IL-32α for stimulating inflammatory cytokines and further confirm the association between IL-32 and IL-6 that we observed *in vivo* in our cohort of SP subjects. The results also suggest a novel role for IL-32 in promoting activation and/or expansion of Th17 cells that are susceptible to HIV infection.

## Discussion

In the current study we showed that among the HIV-infected SPs, EC experienced the lowest rate of CD4^+^ T-cell decline and the lowest frequency of subjects showing loss of virological control. This was further confirmed by setting a threshold of CD4^+^ T-cell decline to 100 cells/mm^3^ combined with increased Log_10_ VL between distal clinic visits with an average time interval of up to 3 years. The EC subgroup compared to VC and NVC showed a statistically significant superiority in maintaining sustained VL and stable CD4 counts. This could not be explained by the distribution and frequency of protective MHC class I antigens such as HLA-B*27 and B*57 within each subgroup, as the between group differences were not statistically significant. However, by comparing the whole SP group to HIV-infected TP, SPs had statistically higher frequencies of protective MHC class I.

Host genetic factors have been implicated in the control of HIV pathogenesis as evidenced by the higher frequency of protective HLA alleles (HLA-B*27, HLA-B*51, and HLA-B*57) in SPs^15^,^16^,^37^. Specifically, in treatment-naive populations, steady-state VL can be in part explained by human genetic variations, with certain single nucleotide polymorphisms (SNPs) at the HLA B and C loci being associated with lower viral loads (VL)^38^. However, the variable disease progression observed among aviremic controllers who maintain undetectable viremia^39^, combined with the lack of disease progression in another subset of subjects with moderate levels of HIV-RNA, would then suggest that clinical progression and viral control may represent distinct phenotypes. In agreement with these observations, our results showed that several SP subjects who lost virological control had at least one HLA protective allele, thus pledging for other detrimental factors to be implicated in the control of disease progression. Among these potential factors, persistent low level of inflammation might be a major drive for disease progression within the different subgroups of SPs^39^. SPs have a low but significant level of immune activation that is likely induced by persistent exposure to HIV. D-dimer, soluble CD163 and lymphoid tissue fibrosis were all reported to be increased in EC subjects^40^. Interestingly, by employing genome-wide transcriptional analysis and *in vitro* and *in vivo* validation of protein expression on primary cells from SPs losing virologic control, we showed that the proinflammatory cytokine IL-32 is modulated at the transcriptional level. Upon loss of control, there was a significant decrease in the less active inflammatory isoforms of IL-32 and, in contrast, an increase in the more proinflammatory isoforms.

IL-32 is a relatively newly identified cytokine that was initially known as the Natural Killer induced transcript 4 (NK4), which is selectively expressed in NK and T-cells upon stimulation with IL-2 and mitogens, respectively^41^. The biological functions of the protein encoded by this NK4 transcript were consistent with that of a cytokine. NK4 was renamed IL-32 following the discovery of its typical proinflammatory features, as it induces a panel of other inflammatory cytokines including TNFα, IL-8, IL-6 and macrophage inflammatory protein-2 (MIP-2)^32^,^33^,^42^. The majority of these functions are mediated by the specific activation of signaling pathways involving p38 mitogen-activated protein Kinase (MAPK) and nuclear factor NF-κB as well as activator protein-1 (AP-1)^23^. Interestingly, IL-32 is expressed in at least 9 different isoforms (α, β, γ, δ, ε, ζ, η, ς and θ) generated by alternative splicing^43^. The γ protein is the longest (full protein) and most active pro-inflammatory isoform and the α isoform is the shortest and least active one^44^. Information on the detailed functions of these individual isoforms and their corresponding receptors and the reason why there are as many as 9 different protein isoforms remain unclear. However, some evidence suggests that splicing of the full γ protein to shorter isoforms represents a safety switch mechanism to protect against uncontrolled and exaggerated inflammation^25^.

IL-32 was initially shown to play a potential antiviral role at the intracellular level, as targeting IL-32 with RNA interference significantly increases HIV replication^23^,^24^. However, RNA interference was not targeting specific IL-32 isoforms but rather the total protein. The antiviral activity of IL-32 in this context was essentially mediated through interferon type I and II and interferon-stimulated genes (ISGs)^23^. Interestingly, the beneficial role of the modest anti-HIV activity of IL-32 was later challenged with *in vivo* data from HIV-infected subjects. Smith *et al*.^31^ clearly showed that IL-32 expression is highly detectable in lymphoid tissues from HIV^+^ subjects representing all stages of infections; acute, asymptomatic and AIDS. In these lymphoid tissues, IL-32 induces the immunosuppressive enzyme indoleamine 2, 3-dioxygenase (IDO) and the negative regulatory protein Ig-like transcript 4 (ILT4) and hence positively correlates with enhanced viral replication and decreased CD4^+^ T-cell survival. This marked effect was shown to be mediated by IL-32γ as shown by their *in vitro* data. This is in line with our current observations that the least active isoform of IL-32 (IL-32α) is indeed decreased and, in contrast, the non-α proinflammatory isoforms are significantly increased in HIV infected SPs experiencing loss of VL control and also from HIV-infected primary cells *in vitro*. Increased protein levels of the non-α IL-32 isoforms was evidenced by the detection of increased levels of total IL-32 using antibodies having the capacity to recognize the four major isoforms (α, β, γ, δ) meanwhile a decrease in the protein levels of the α isoform (detected with α-specific antibodies). As our transcriptomic data on subjects with control failure also showed a decrease in the IL-32δ isoform, the aforementioned increase in total IL-32 is high likely due to increased levels of IL-32 β and γ. However, the relative contribution of each of these two isoforms in the increased levels of IL-32 upon loss of control was not determined in the current study due to technical difficulty. Our observations on the abundance of the proinflammatory non-α isoforms of IL-32 in HIV-infected subjects compared to HIV-negative donors, who in contrast have higher levels of the less inflammatory isoform α, are consistent with earlier reports on the differential expression of IL-32 isoforms under inflammatory conditions^45^. However, a study by Monteleone *et al*.^46^ reported increased expression of all IL-32 isoforms including both α and non-α isoforms, in HIV-infected compared to HIV-negative individuals. Although these studies were carried out only on the transcriptional level (mRNA) without validating the protein expression of the different isoforms of IL-32, they might suggest that post-transcriptional modifications of isoform expression may take place in HIV infection to favor the expression of the non-α isoforms. Further studies at the molecular level are then warranted to understand the underlying mechanisms of differential IL-32 isoform expression upon loss of HIV control and increased viral load. In this regard, it remains unclear how HIV modulates the transcription of IL-32 to increase the pro-inflammatory and decrease the less inflammatory isoforms. It is also unclear how and when these proinflammatory isoforms drive loss of control in HIV-infected subjects with history of disease control. This remains an open question that we will tackle in future studies measuring longitudinally the modulation of IL-32 expression from these subjects.

Sustained inflammatory environment is likely to be advantageous for HIV replication. Our *in vitro* data showing activation of CD4^+^ T-cells by IL-32γ suggest that IL-32 might enhance HIV replication. In this regard, IL-32γ induced high levels of both IL-17 A and IL-17 F, two cytokines produced by Th17 CD4^+^ T-cells, which are major players in mucosal immunity and that are depleted in HIV infection^34^,^47^,^48^. These data are in line with previous reports linking IL-32γ inflammation with increased Th1 cytokines and induction of Th17 polarizing cytokines by phospholipase C/JNK and NF-κB dependent mechanisms^49^. Interestingly, by using *Gene Set Variation Analysis* (GSVA)^50^ on our microarray data from subjects losing control, we identified an increase in the inflammasome and loss of Th17 pathways as a top hit (data not shown). This may suggest that sustained inflammation and activation of Th17 cells by IL-32 and potentially productive HIV infection may contribute to a significant depletion of these cells *in vivo*. Th17 cells play a central role in mucosal immunity and depletion of these cells underlies the marked mucosal insult, microbial translocation (MT) and associated pathologies in HIV-infected subjects^34^,^51^,^52^. On its turn, MT is associated with systemic circulation of different bacterial products, including lipopolysaccharides (LPS), leading to chronic innate immune activation (CI)^53^,^54^,^55^,^56^. One of the major consequences of CI is the persistent activation of monocytes by LPS^56^. Monocytes actively respond to LPS by up-regulating and releasing the LPS co-ligand, the soluble CD14 (sCD14)^57^. Interestingly, our studies showed that increased IL-32 levels in SP subjects is associated with increased sCD14 and further links this to the activation and potential depletion of Th17 cells. More importantly, our results show a strong predictability of both inflammation (as shown by IL-6 and CD4/CD8 ratio) and sCD14 levels by IL-32 over periods that ranged from few months to few years, thus placing IL-32 upstream of these pathways. This is further supported by the strong correlation but also the predictability of CD4 decline and increased VL by total IL-32 levels in the periphery of SP subjects. Deterioration of these two major clinical factors is then likely linked to the IL-32-mediated inflammation and pathology (impact on Th17 cells) and suggests circulating IL-32 as a potential therapeutic target.

In summary, our current report highlights an unappreciated role for the recently described cytokine IL-32 in disease progression in HIV-infected SPs. IL-32 significantly correlates with the loss of virological control and CD4 decline in these SP subjects and furthermore could reliably predict this loss of control over a period of time that ranged from few months to several years. Our proof-of-concept for the predictability of disease progression by the plasma levels of IL-32 then warrants further investigations in the general population of HIV-infected subjects including those under treatment. We also suggest that IL-32 may represent a potential therapeutic target to prevent persistent inflammation and its associated pathologies in the HIV-infected population.

## Methods

### Study cohort

Our study is focused on the CCHSP that was launched in 2005 and is currently enrolling treatment-naive study participants from 18 centers across Canada. HIV infected SPs, defined according to the characteristics presented in Table 1, were enrolled to the study following written informed consent if they met the following inclusion criteria: 1) HIV positive by ELISA with a confirmed Western Blot 2) 18 years and older 3) had never received ART. In addition, a treatment-naive “control” cohort was simultaneously enrolled during HIV primary infection and followed up to 2 years.

Demographic and disease history data collected at baseline included age, sex, race, mode of transmission, age at diagnosis, date of diagnosis and number of years infected at baseline, and information on HIV risk behaviors and drug use. Baseline assessments included a physical exam signs and symptoms of HIV infection. In addition, the disease trajectory for each study participant was also established using abstracted data on all CD4 counts and HIV-RNA VLs from the medical charts. Four weeks after the baseline evaluation, study participants provided a medical history update and underwent routine biochemistry and hematology blood work and CD4^+^ and CD8^+^ T cell counts, including subset percentages and provided samples for HIV-1 DNA assays. Finally, 135 mL of whole blood was collected at the one month assessment to conduct genetic analyses for genetic polymorphism in genes associated with slow disease progression, including MHC class I HLA- B*14, -B*27, -B*38, -B*51, -B*57, -B*58, -B*81^16^,^17^,^18^. Genotyping was done as previously described^58^. At each subsequent follow up visit, study participants underwent a routine physical exam and provided blood specimens for routine chemistry and hematology and study-specific CD4^+^ T-cells and HIV-RNA assays. Study participants were seen at baseline and then at months 1, 6, 7 and 12 during the first year of the study. Participants continued to be seen according to the same schedule (twice every six months, one month apart) for a total of five years. Study participants were then re-consented to continue to be followed under an optional extension study.

Study participants were excluded from the study if they chose to initiate ART, failing to keep appointments or missing 2 study visits in a row or if they wished to be removed from the study. Subjects necessitating ART initiation as indicated by their physician, were maintained in the study. In study participants receiving ART, clinical and laboratory data continued to be collected according to the study schedule, however research blood draws were only performed once a year.

Data collected prospectively as part of this study was then combined with historical data from the medical charts at the time of enrolment to establish the disease trajectory for each study participant. Disease progression was defined as a significant negative CD4 slope combined with a significant positive VL slope calculated on historical and prospective clinical records since the time of infection for each subject. Loss of control between visits was calculated for each subject as having a loss of CD4 counts ≥ 100 cell/mm^3^ combined with any increase in the Log_10_ VL.

### Study approval

This study was approved by the Institutional Review Boards (IRB) of the Centre Hospitalier de l’Université de Montréal Research Center and at all participating sites’ IRBs. All experiments were performed in accordance with the guidelines and regulations approved by the ethic committees from CRCHUM and all IRBs (Ethical approval #SL 04–061). Study participants provided written informed consent for the use of their plasma and cells for the current research investigation.

### Transcriptomic analysis by microarray

Total cellular RNA was extracted from PBMC of selected HIV-infected SPs using the RNeasy columns kit (Qiagen Cat # 74104), according to the manufacturer’s directions, followed by quality assessment and quantification. RNA reverse transcription and gene expression profiling from V1 and V2 (before and after loss of control) from n = 5 SP subjects was carried out on the microarray platform of the Génome Québec Innovation Center (Montreal, QC, Canada). The platform uses the Illumina HumanHT-12 v4 Expression BeadChip that provides more than 47,000 transcripts and known splice variants across the human transcriptome. Data generated by the Illumina Bead arrays were first inspected for errors followed by a series of exploratory data analysis methods that are used to identify the potential sources of expression variation or bias (PCA/ICA, MDS, SVA), and identification of potential technical or biological outliers (hierarchical clustering). Proper normalization or transformation of the data was customized based on the observed results and needs. For differential gene expression, moderated t-statistics were calculated using multiple regressions with the inclusion of known covariates or new ones identified in the exploratory phase. This analysis was performed with the Limma software suite^59^. Differentially expressed genes with a cut-off value greater than 1.3-fold and a statistical p value < 0.05 were considered for further analysis. The gene networks for candidates of interest were generated through the use of Ingenuity Pathways Analysis (Ingenuity^®^ Systems, www.ingenuity.com) and *Gene Set Variation Analysis* (GSVA).

### HIV VL, absolute T-cell counts and genetic polymorphism determinations

Blood samples were collected in EDTA tubes from study participants undergoing routine laboratory measures. Absolute CD4 and CD8 T-cell counts were obtained using BD FACSCount reagent Kits according to manufacturer’s directions (standardized clinical measures). The Antibody mixture for absolute counts contained custom-made antibodies specific for CD3, CD4, CD8, CD19 (to exclude B cells), CD56 (to exclude NK cells), CD45RA and CD38 (for cell activation profile). HIV plasma VL was determined by the ultrasensitive assay Amplicor (Roche) with a range of amplification of 50–750,000 copies/ml.

### Measurements of soluble proteins and cytokines

Plasma samples from HIV-infected and HIV^neg^ subjects were used to quantify human soluble CD14 (sCD14) using Quantikine ELISA Kit (R&D Systems, Cat # DC140), total IL-32 by DuoSet Human IL-32 (R&D Systems, Cat # DY3040), IL-32 alpha isoform using competitive Human Interleukin 32α (IL-32α) Elisa kit (MyBioSource, Cat # MBS750027), IL-6 using Human IL-6 ELISA Ready SET-Go (eBioscience, Cat # 88-706622), according to the supplier’s protocol. Cell-associated total IL-32 was measured by ELISA from cell lysates prepared as follows: total PBMCs from HIV^+^ or HIV^neg^ subjects were lysed by 1X RIPA buffer (Cell Signaling, Cat #9806) supplemented with protease inhibitors (Roche, Cat # 04 693 159 001 and 04 906 837 001) followed by quantification of total protein *per* lysate using the Bradford assay^60^. ELISA measures from total cell lysate were normalized to the total protein in the lysate and plotted on graphs as IL-32 pg *per* 1μg of total cellular protein.

### Cell purification, stimulation and infection with HIV-1

CD4^+^ T-cells were isolated from total PBMCs by negative selection using EasySep Human CD4 T Enrichment columns (StemCell, Cat #19052). Purity of CD4 was typically >98% as determined by surface staining of CD4^+^ T-cells with CD3-Pacific Blue (UCHT1) and CD4-Alexa Fluor 700 (RPA-T4), (both from BD) and FACS analysis (BD LSRII analyser). Purified CD4^+^ T-cells were stimulated with plate-coated anti-CD3 antibody (0.5μg/ml) and soluble anti-CD28 antibody (0.5μg/ml) (both from BD Biosciences, Cat ##555329, 5555726 respectively) for 48 h in the presence or absence of 500 ng IL-32α (BioLegend, Cat # 551004) or IL-32γ (R&D Cat # 4690-IL-025/CF). Secreted cytokines were measured from the supernatant of activated cells using the LEGENDplex™ Human Th Cytokine Panel (13-plex) (BioLegend, Cat # 740001) according to manufacturer’s directions. Infections of PBMCs stimulated with PHA and IL-2 (0.25μg/ml, and 100 units/ml, respectively) or resting cells were carried out using 50 ng of the laboratory strain HIV-BaL *per* 10^6^ cells by Spinoculation^61^ (2 hours/Room temperature). Following infection, PBMCs were washed twice by PBS and centrifuged at 1500 rpm for 5 min then were re-suspended in RPMI-1640 medium supplemented with 10% FBS. Cells were kept in culture for 48 h before collection of supernatants and cells for cytokine measures.

### Statistical analysis

Data were analyzed using GraphPad Prism 4 (GraphPad software, San Diego, CA). Data from the same subjects before and after observation were analyzed using Wilcoxon matched pairs test. Comparison between 3 or more groups for the same observation was done with the non-parametric Kruskal-Wallis test followed by Dunn’s post tests to investigate difference between paired groups. Differences between two groups of subjects for the same observation was done using the non-parametric Mann-Whitney. Any other methods are described as appropriate in the text.

## Additional Information

**Accession numbers**: The entire microarray dataset and technical information requested by Minimum Information About a Microarray Experiment (MIAME) are available at the Gene Expression Omnibus (GEO) database under accession number GSE74790.

**How to cite this article**: El-Far, M. *et al*. Proinflammatory isoforms of IL-32 as novel and robust biomarkers for control failure in HIV-infected slow progressors. *Sci. Rep.* **6**, 22902; doi: 10.1038/srep22902 (2016).

## Supplementary Material

Supplementary Information

## Figures and Tables

**Table 1:** Classification and clinical characteristics of HIV-infected slow progressor subgroups.

Slow Progressor subgroups/number of subjects^A^	CD4^+^T-cell count at baseline	HIV-RNA viral load (VL) at baseline	Time since infection	ART^B^
Elite controller (n = 45)	>500 cells mm^3^	≤50 copies/ml	Any	NO
Virologic controller (n = 68)	>500 cells mm^3^	51–3000 copies/ml	Any	NO
Non-Virologic controller(n = 33)	>500 cells mm^3^	>3000 copies/ml	>7 years	NO

^A^Slow progressor: HIV^+^ study participant meeting any of shown definitions and having no signs of AIDS.

^B^Antiretroviral treatment.

**Table 2:** Proportion of subjects with different HLA types.

All SP	Elite (n = 42)	Virologic (n = 63)	Non-virologic (n = 30)	P value^A^	All SP (n = 135)	Primo(n = 455)	P value^B^
Num of protective alleles, n (%)				0.525			<0.001
0	13 (31.0)	22 (34.9)	15 (50.0)		50 (37.0)	261 (57.4)	
1	22 (52.4)	33 (52.4)	12 (40.0)		67 (49.6)	180 (39.6)	
2	7 (16.7)	8 (12.7)	3 (10.0)		18 (13.3)	14 (3.1)	
With protective alleles, n (%)				0.229			<0.001
No	13 (31.0)	22 (34.9)	15 (50.0)		50 (37.0)	261 (57.4)	
Yes	29 (69.0)	41 (65.1)	15 (50.0)		85 (63.0)	194 (42.6)	
HLA type, n (%)							
B*13	2 (4.8)	5 (7.9)	3 (10.0)	0.702	10 (7.4)	8 (1.8)	0.001
B*14	3 (7.1)	10 (15.9)	6 (20.0)	0.258	19 (14.1)	43 (9.5)	0.124
B*27	12 (28.6)	11 (17.5)	1 (3.3)	0.022	24 (17.8)	37 (8.1)	0.001
B*38	1 (2.4)	0 (0.0)	0 (0.0)	0.533	1 (0.7)	18 (4.0)	0.063
B*51	4 (9.5)	5 (7.9)	1 (3.3)	0.640	10 (7.4)	65 (14.3)	0.035
B*57	11 (26.2)	15 (23.8)	4 (13.3)	0.397	30 (22.2)	25 (5.5)	<0.001
B*58	3 (7.1)	2 (3.2)	2 (6.7)	0.604	7 (5.2)	12 (2.6)	0.141
B*81	0 (0.0)	1 (1.6)	1 (3.3)	0.492	2 (1.5)	0 (0.0)	0.052
3DL1*h/*y + B*57	3 (7.9)	8 (14.0)	3 (10.3)	0.717	14 (11.3)	9 (2.0)	<0.001

Genotype data missing for 11 SP subjects and 35 subjects in the Primo cohort

Protective alleles - among HLA-B*13, B*14, B*27, B*38, B*51, B*57, B*58 and B*81

P values are based on Fisher’s exact test/Chi square test as appropriate

^A^Comparison between the SP.

^B^Comparison between all SP and Primo.

**Table 3:** Rate of CD4 decline and increased viral load in subjects falling control.

Subject ID	Time Since infection (Years)	CD4 decline (Cells/mm^3^/year ± SD)	P value	Viral load increase (HIV copies/year ± SD)	P value
EC					
218001	5.19	−88.59 ± 40.19	0.0478	25.41 ± 8.83	0.0139
VC					
102018	14.74	−24.92 ± 3.40	<0.0001	55.37 ± 25.86	0.0393
104001	7.55	−37.67 ± 11.67	0.008	253.6 ± 39.98	<0.0001
104002	11.41	−33.10 ± 7.36	0.0001	3355 ± 473.2	<0.0001
104003	22.29	−21.52 ± 3.76	<0.0001	3350 ± 748.3	0.0012
110004	15.78	−31.21 ± 3.45	<0.0001	233.5 ± 61.59	0.001
205002	6.59	−33.80 ± 14.10	0.031	919.5 ± 230.4	0.0013
501008	6.64	−49.36 ± 16.05	0.0077	163.6 ± 60.05	0.0164
NVC					
104008	13.37	−28.64 ± 4.76	<0.0001	11270 ± 3028	0.0008
105003	10.3	−44.14 ± 8.13	<0.0001	21120 ± 7188	0.0056
106006	18.28	−30.46 ± 3.91	<0.0001	3952 ± 438.8	<0.0001
108001	10.51	−74.19 ± 13.27	0.0001	7022 ± 2666	0.0218
109003	13.25	−18.45 ± 4.22	0.0002	1467 ± 335.5	0.0002
109007	17.1	−23.41 ± 3.81	<0.0001	5589 ± 958.2	<0.0001
110001	13.39	−69.76 ± 9.35	<0.0001	2780 ± 652.9	0.0004
110005	15.6	−31.19 ± 4.30	<0.0001	1609 ± 443.1	0.0015
401010	8.24	−64.35 ± 14.02	0.0001	4918 ± 1588	0.0045
