# Supplementary material for: Proinflammatory isoforms of IL-32 as novel and robust biomarkers for control failure in HIV-infected slow progressors
Source: Sci Rep. 2016 Mar 15;6:22902. doi: 10.1038/srep22902 (PMC4792165; doi:10.1038/srep22902)
Supplement: Supplementary Information [file srep22902-s1.pdf]

Proinflammatory isoforms of IL-32 as novel and robust biomarkers for control failure in HIV-infected slow progressors

Mohamed El-Far[1]*, Pascale Kouassi[1,2], Mohamed Sylla[1], Yuwei Zhang[1,2], Ahmed Fouda[1], Thomas Fabre[1,2], Jean-Philippe Goulet[3], Julien van Grevenynghe[4], Terry Lee[5], Joel Singer[5], Marianne Harris[6], Jean-Guy Baril[7], Benoit Trottier[8], Petronela Ancuta[1,2], Jean-Pierre Routy[9], Nicole Bernard[10] and Cécile L. Tremblay[1,2]* for the Investigators of the Canadian HIV$^+$ Slow Progressor Cohort.

Supplementary information

Supplementary Figure legends

Supplementary Figure S1: Impact of gender (a) and age (b) on the decline of $CD4^+$ T-cell counts in the different slow progressor (SP) subgroups. Mixed effects regression analysis on CD4 decline for Elite controllers (n=45) (Left upper panel), Virologic controllers (n=68) (Right upper panel), Non-virologic controllers (n=33) (Left lower panel) compared to typical progressors (Primo) (n=490) (Right lower panel). NS= non-significant.

Supplementary Figure S2: Impact of race on the decline of $CD4^+$ T-cell counts in the different slow progressor (SP) subgroups. Mixed effects regression analysis on CD4 decline for Elite controllers (n=45) (Left upper panel), Virologic controllers (n=68) (Right upper panel), Non-virologic controllers (n=33) (Left lower panel) compared to typical progressors (Primo) (n=490) (Right lower panel). NS= non-significant.

Supplementary Figure S3: Impact of sexual *versus* non-sexual (a) and intravenous drug use (b) on the decline of $CD4^+$ T-cell counts in the different slow progressor (SP) subgroups. Mixed effects regression analysis on CD4 decline for Elite controllers (n=45) (Left upper panel), Virologic controllers (n=68) (Right upper panel), Non-virologic controllers (n=33) (Left lower panel) compared to typical progressors (Primo) (n=490) (Right lower panel). NS= non-significant.

Supplementary Figure S4: Transcriptional analysis by microarray. Shown is a heatmap for top differentially expressed genes including the IL-32α ($p<0.05$, fold change with a cut-off of 1.3 between V1 and V2 for each of 5 subjects who lost HIV control).

Supplementary Figure S5: Cell-associated total IL-32. (**a**) Total cell protein from PBMCs of HIV^{neg} (n=7), EC (n=10) and TP (n=9) was determined following cell lysis and used to measure total IL-32 by ELISA. Shown are the levels of IL-32, in 1μg of total cell protein. (**b**) Correlations between the levels of plasma and cell-associated total IL-32 from the same subjects (EC, n=8 and TP, n=9). Kruskal-Wallis and Dunn's post tests were used to assess the significance of between-group differences in panel A. Spearman correlation tests were used to assess the significance of the correlation between cells associated and plasma total IL-32 from the same subjects. ρ= correlation coefficient. P-values over lines linking 2 data sets refer to comparisons of these 2 groups.

Supplementary Figure S6: Correlations between sCD14 and $CD4^+$ T-cell counts (**a**), Log_{10} VL (**b**) and CD4/CD8 ratio (**c**) measured in plasma from the same EC (n=19), VC (n=22), NVC (n=6) subjects at V1 and V2. A Spearman correlation test was used to assess the significance of correlations between the 2 measured parameters. The correlation coefficient (ρ) and p-value for each comparison are shown over the graphs.

Supplementary Figure S7: Correlations between IL-6 and $CD4^+$ T-cell counts (**a**), Log_{10} VL (**b**) and CD4/CD8 ratio (**c**) measured in plasma from the same EC (n=19), VC (n=22), NVC (n=6) subjects at V1 and V2. A Spearman correlation test was used to assess the significance of correlations between the 2 measured parameters. The correlation coefficient (ρ) and p-value for each comparison are shown over the graphs.

Supplementary Figure S8: Correlation between IL-32 levels in plasma at V1 from EC (n=19), VC (n=22), NVC (n=6)) and (**a**) sex (Men n=36, Women n=11), (**b**) age (yrs), (**c**) time between V1 and V2 (months). A Mann-Whitney test was used to assess the significance of between-group differences in

panel A and Spearman correlation test (ρ) was used to assess the significance of correlations between the 2 measured parameters.

Supplementary Figure S1

a)

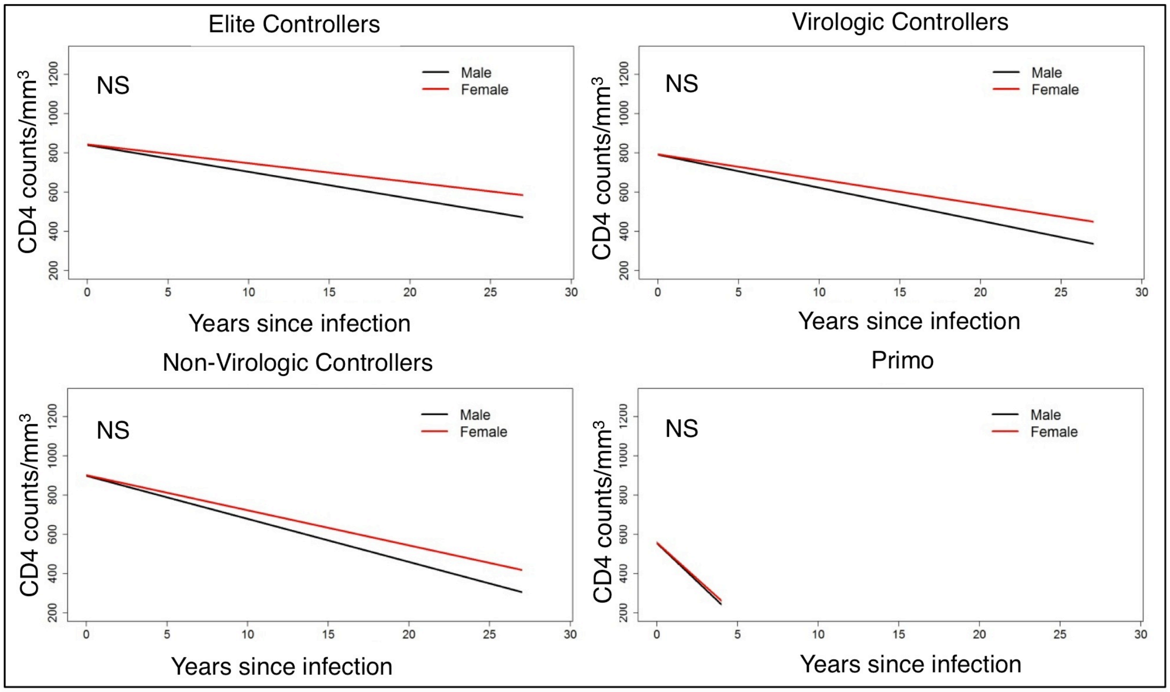

b)

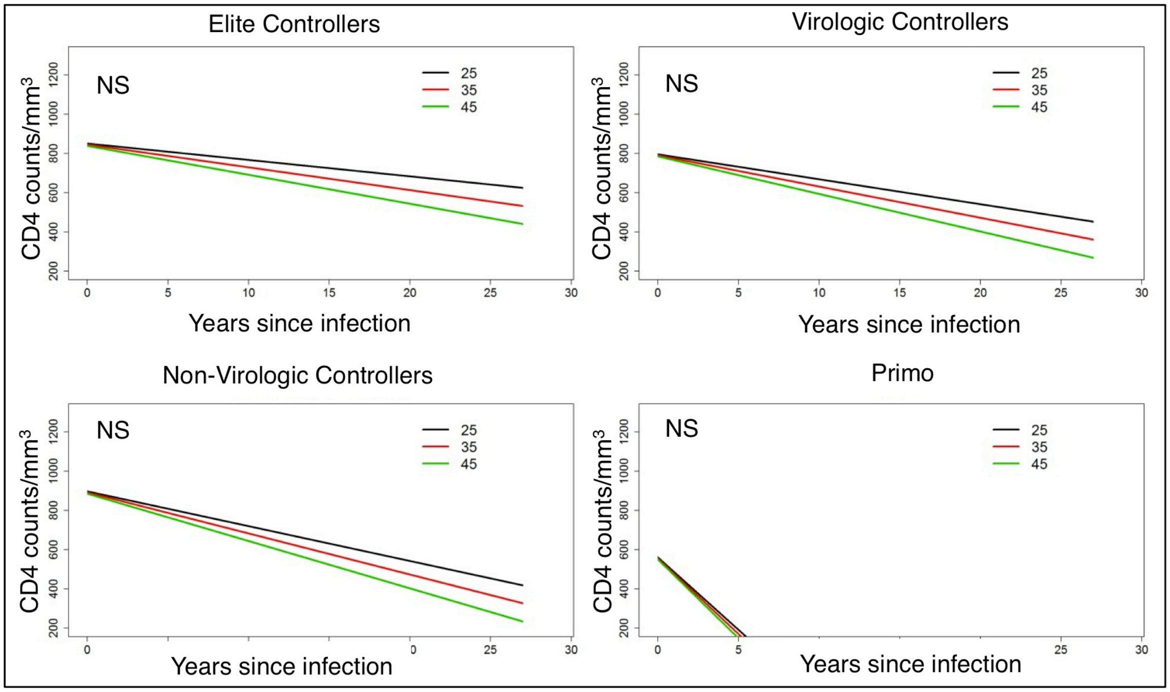

Supplementary Figure S2

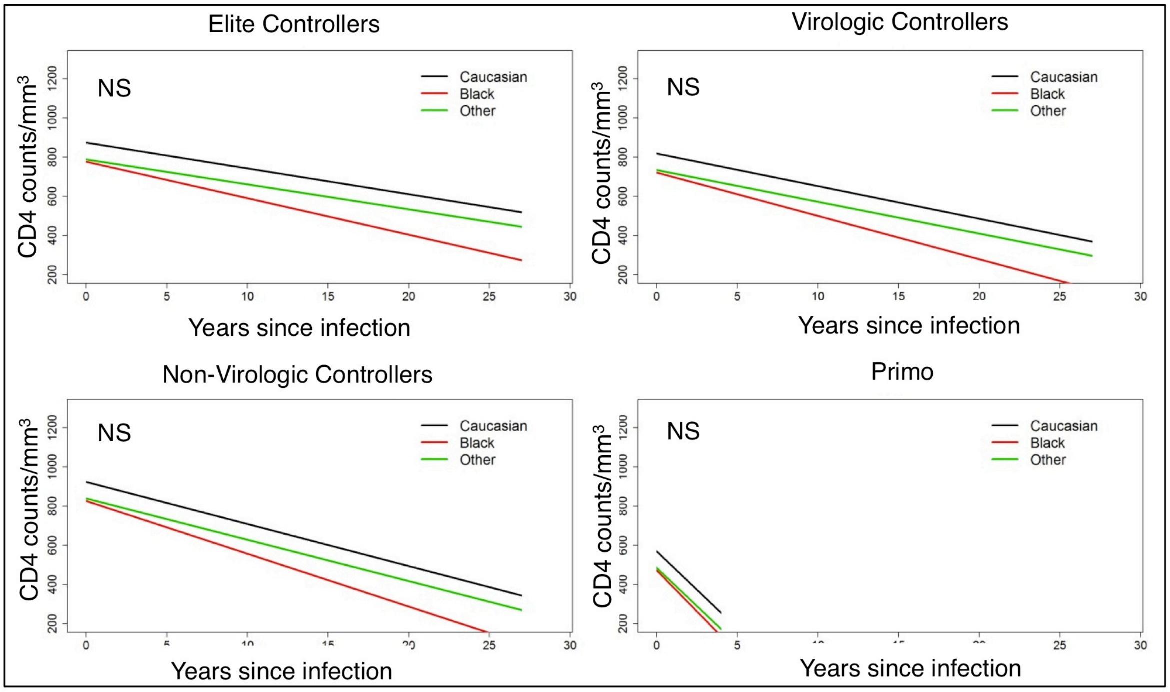

Supplementary Figure S3

a)

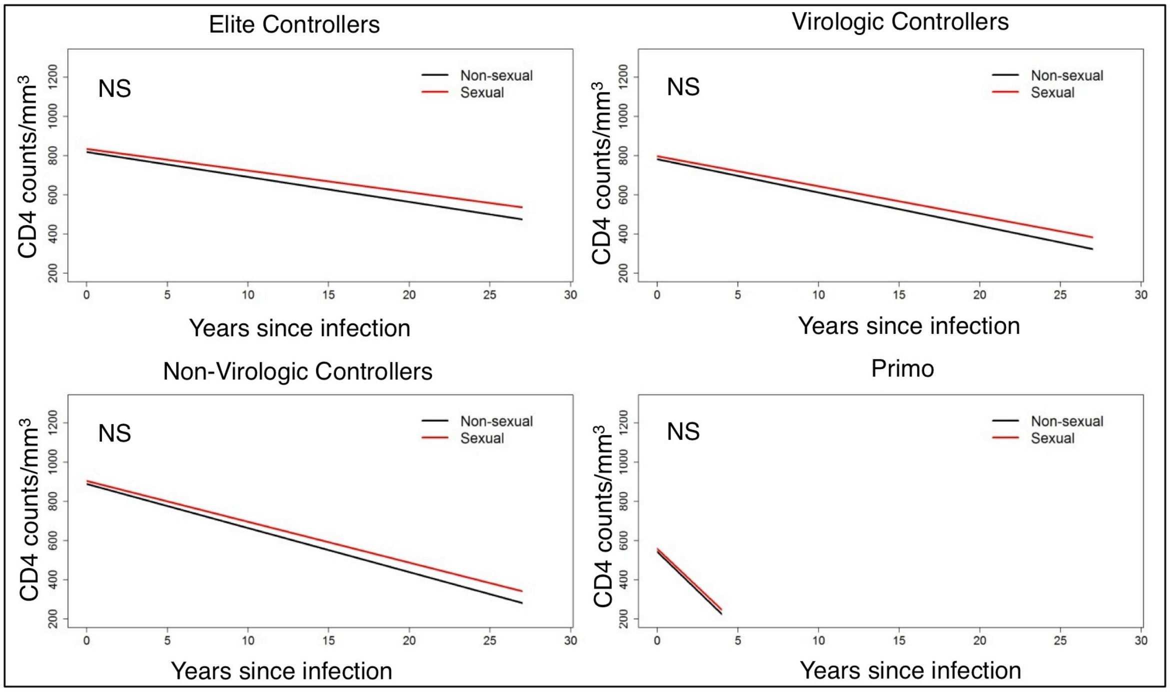

b)

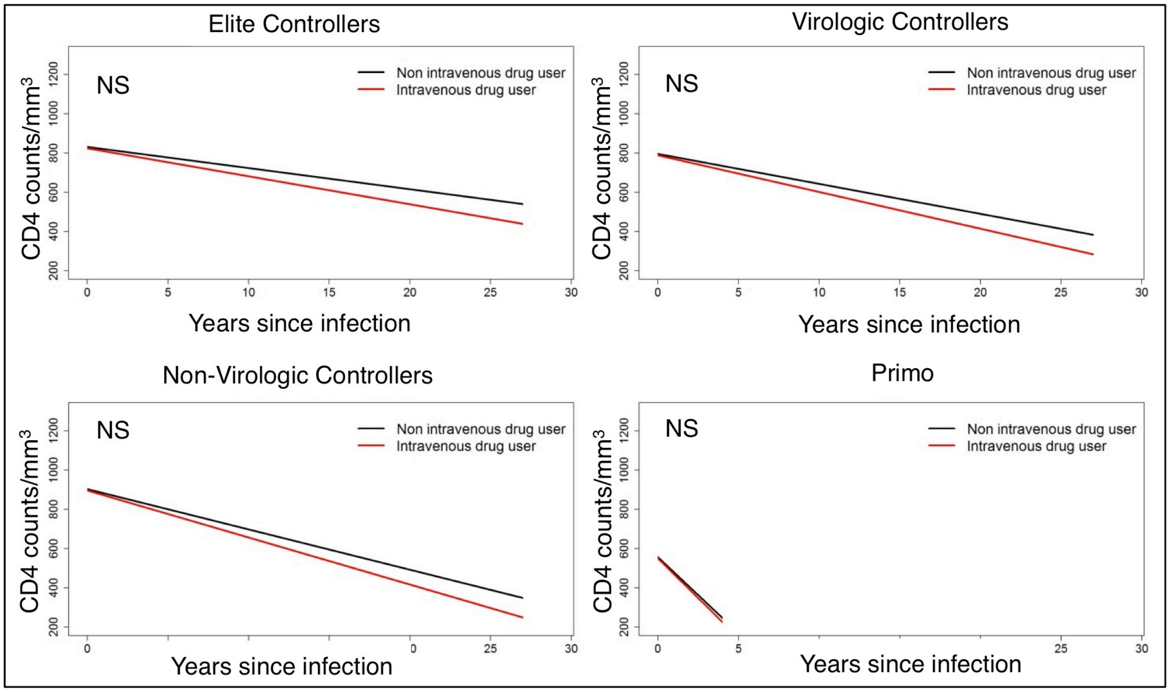

Supplementary Figure S4

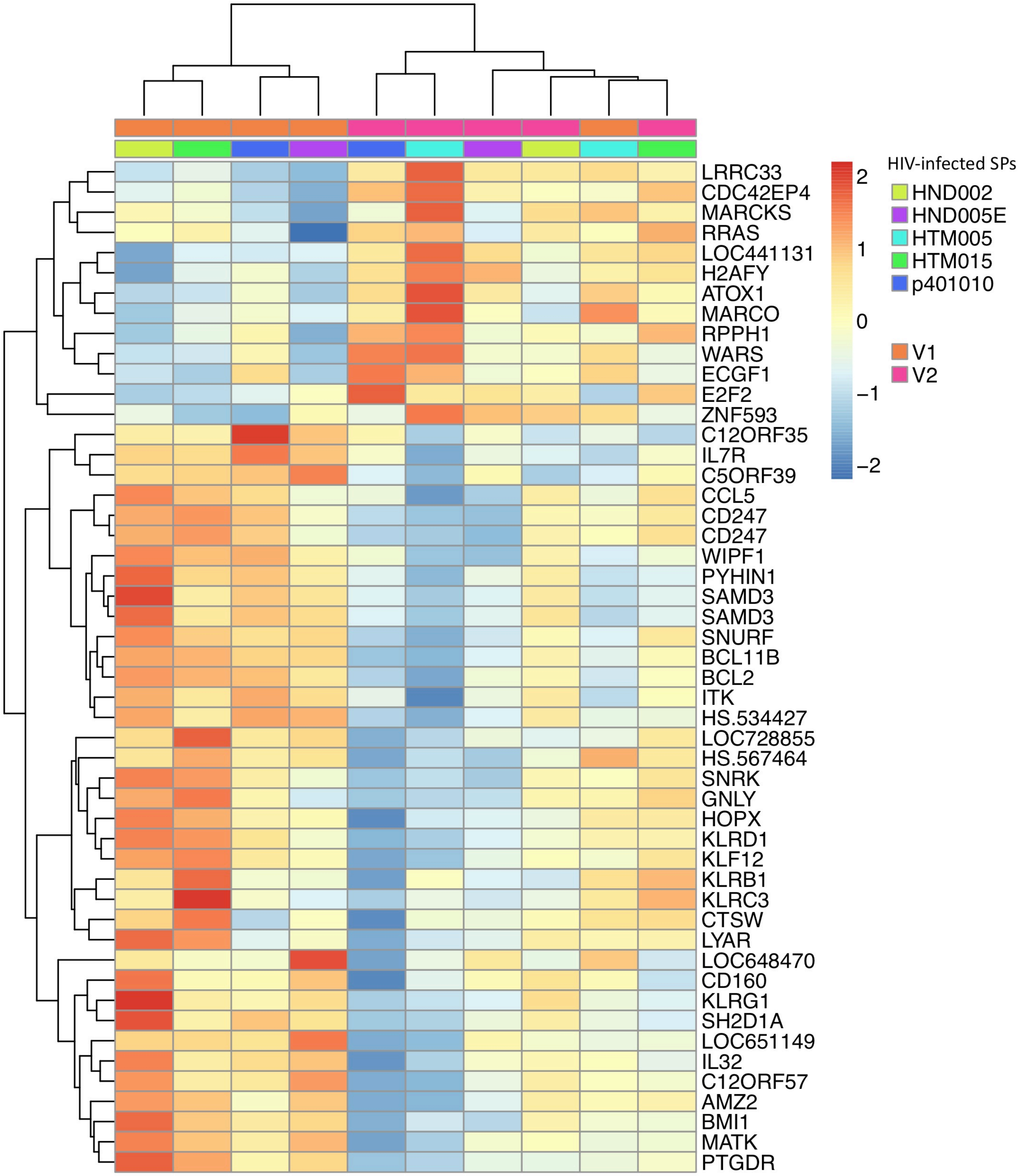

Supplementary Figure S5

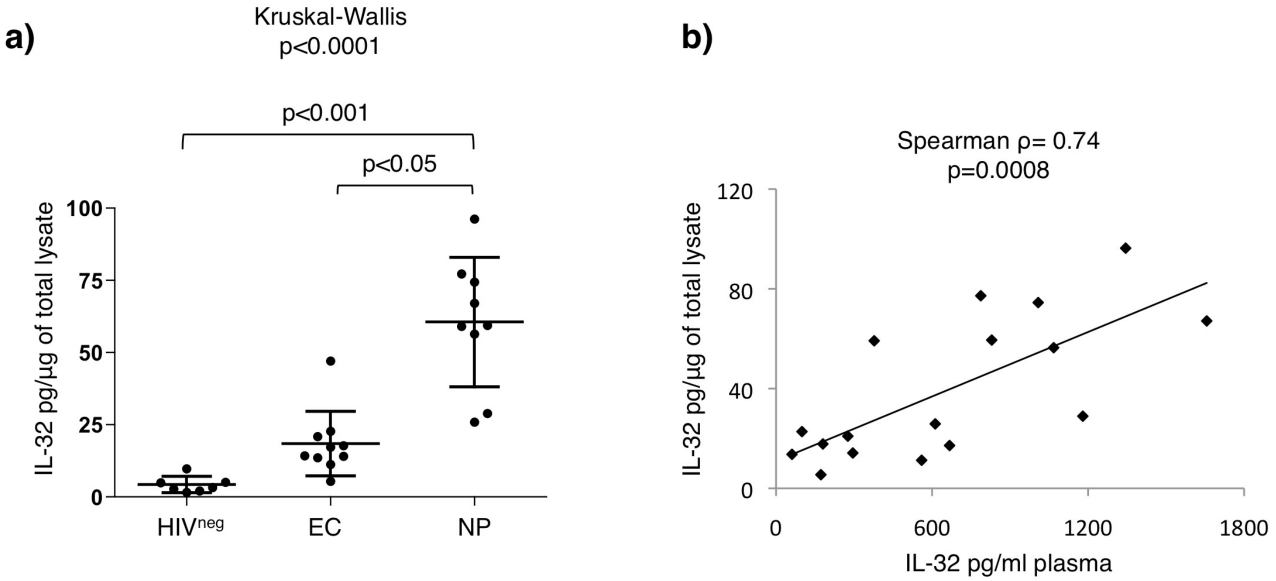

Supplementary Figure S6

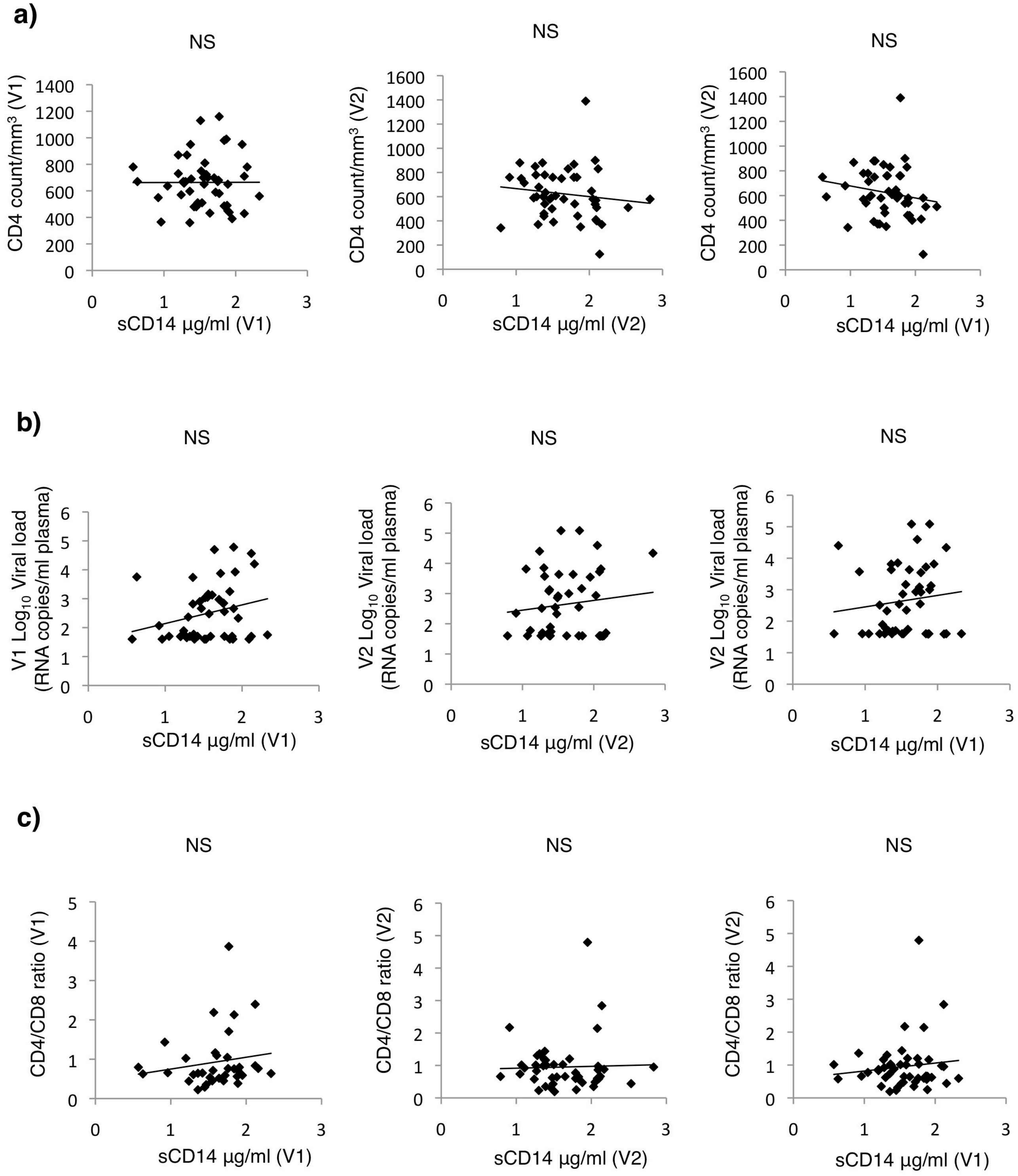

Supplementary Figure S7

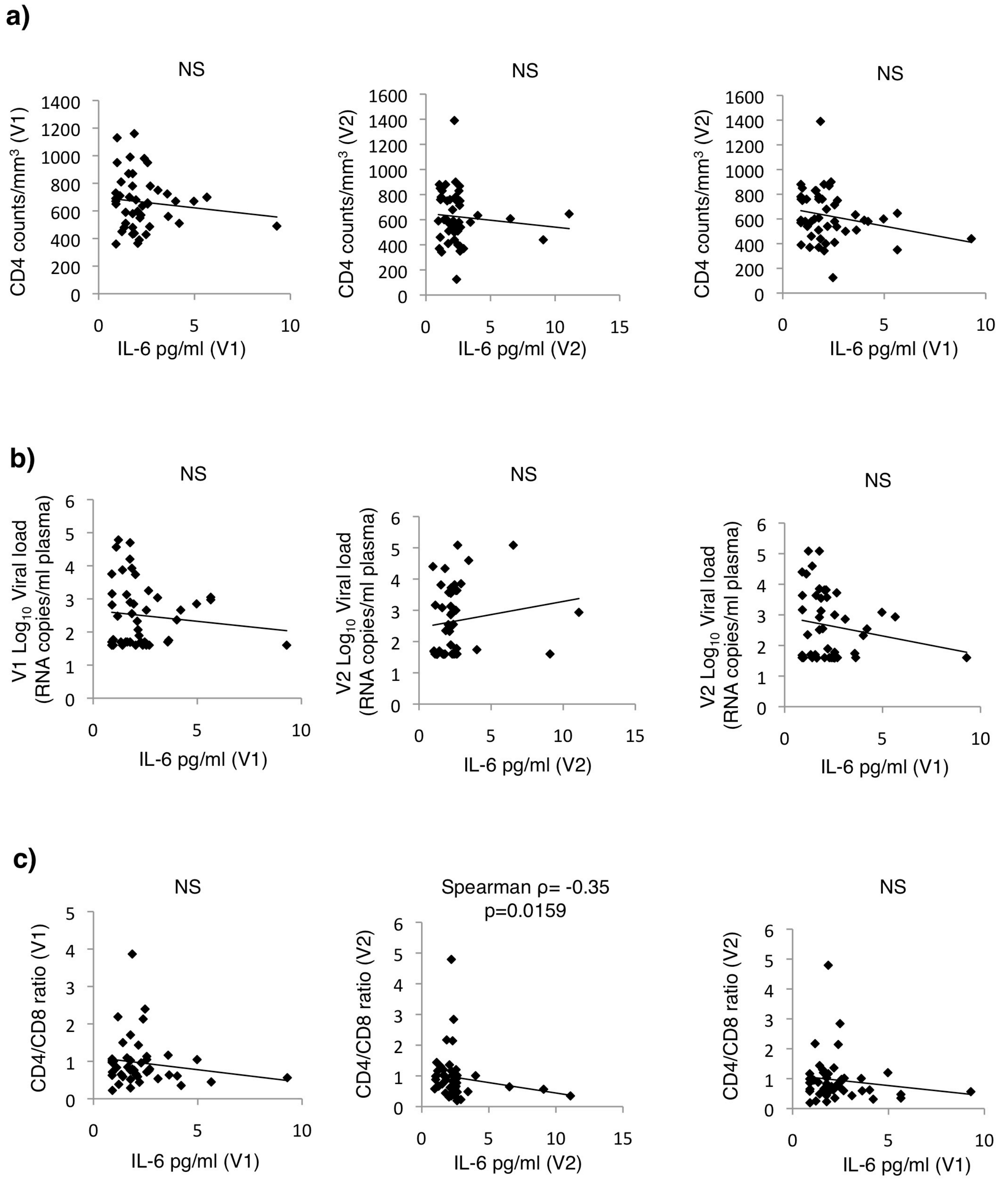

Supplementary Figure S8

a)

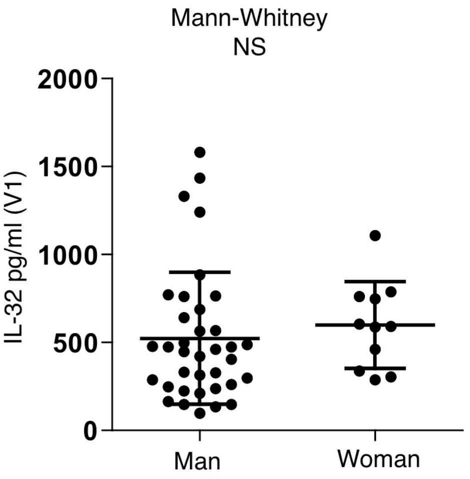

b)

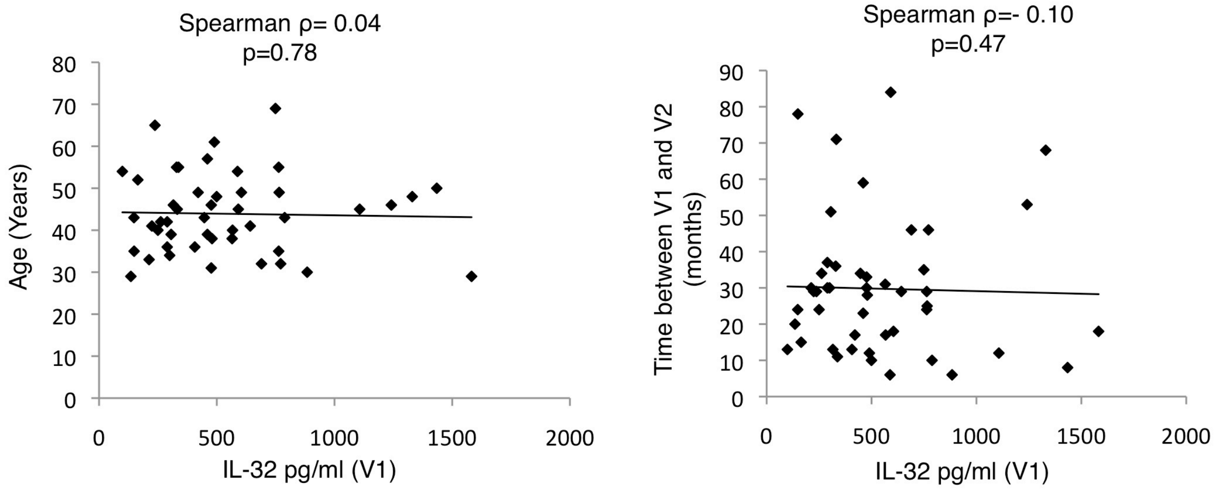

Supplementary Table S1: Demographic and disease history characteristics of the study subjects.

Variable	All patients (n=636)	Slow progressors			PRIMO cohort (n=490)
		Elite (n=45)	Virologic (n=68)	Non-virologic (n=33)	
Sex - male, n (%)[A]	557 (87.6)	31 (68.9)	48 (70.6)	26 (78.8)	452 (92.2)
Race, n (%)					
Unknown	138 (21.7)	3 (6.7)	1 (1.5)	4 (12.1)	130 (26.5)
Caucasian	414 (65.1)	28 (62.2)	46 (67.6)	22 (66.7)	318 (64.9)
Black	42 (6.6)	9 (20.0)	10 (14.7)	5 (15.2)	18 (3.7)
Other	42 (6.6)	5 (11.1)	11 (16.2)	2 (6.1)	24 (4.9)
Modes of transmission, n (%)					
Unknown	17 (2.7)	2 (4.4)	1 (1.5)	1 (3.0)	13 (2.7)
Sexual	529 (83.2)	35 (77.8)	60 (88.2)	29 (87.9)	405 (82.7)
Intravenous drug user	85 (13.4)	6 (13.3)	5 (7.4)	4 (12.1)	70 (14.3)
Other	77 (12.1)	19 (42.2)	34 (50.0)	22 (66.7)	2 (0.4)
Age at diagnosis					
Unknown, n (%)	26 (4.1)	2 (4.4)	1 (1.5)	3 (9.1)	20 (4.1)
Median (IQR)	35.1 (28.4, 42.3)	35.4 (26.4, 43.9)	34.8 (27.4, 39.8)	33.5 (27.3, 44.4)	35.5 (28.8, 42.4)
Years infected at baseline[B]					
Unknown, n (%)	75 (11.8)	0 (0)	0 (0)	0 (0)	75 (15.3)
Median (IQR)	0.4 (0.2, 0.9)	7.4 (2.9, 17.5)	6.1 (3.1, 10.3)	8.8 (5.3, 15.5)	0.3 (0.2, 0.5)
Age at baseline					
Unknown, n (%)	78 (12.3)	2 (4.4)	1 (1.5)	3 (9.1)	72 (14.7)
Median (IQR)	38.2 (30.6, 44.8)	46.0 (38.5, 54.1)	41.4 (34.3, 48.4)	46.1 (34.0, 55.3)	36.1 (29.0, 42.7)
CD4 count at baseline					
Unknown, n (%)	85 (13.4)	0 (0)	0 (0)	0 (0)	85 (17.3)
Median (IQR)	531 (406, 690)	730 (620, 928)	652 (550, 745)	650 (554, 740)	484 (380, 630)
Follow up time, years					
Median (IQR)	0.7 (0.1, 2.0)	7.6 (3.7, 13.0)	7.8 (5.1, 11.8)	10.2 (6.0, 14.0)	0.3 (0.0, 1.5)

Percentages are relative to all subjects within each group, including the ones with missing data
[A]Data missing for 4 SP subjects and 10 PRIMO subjects
[B]For Primo cohort, baseline is defined as the time of the first available data point

Supplementary Table S2: HLA typing for slow progressor subjects experiencing loss of control.

Subject ID/ subgroup	HLA-A	HLA-A	HLA-B	HLA-B	HLA-C	HLA-C	Bw	Bw
Subjects showing loss of control								
218001/EC[1]	A*02	A*03	B*07	B*48	C*07	C*08	Bw6	Bw6
109020/EC	A02	A*11	B*08	B*56	Cw*01	Cw*07	Bw6	Bw6
104002/VC[2]	A02	A*02	B*15	B*44	Cw*02	Cw05	Bw4	Bw6
102018/VC	A*01	A*68	B*57	B*73	C*15	C*17	Bw4	Bw6
109002/VC	A02	A*02	B*27	B*40	Cw*01	Cw*03	Bw4	Bw6
216009/VC	A*02	A*03	B*51	B*52	C*12	C*15	Bw4	Bw4
218004/VC	A*03	A*03	B*07	B*07	C*07	C*15	Bw6	Bw6
205002/VC	A*03	A*68	B*14	B*44	C*08	C*14	Bw6	Bw4
110004/VC	N/A	N/A	N/A	N/A	N/A	N/A	N/A	N/A
109006/VC	A*01	A*11	B*13	B*57	Cw*03	Cw*06	Bw4	N/A
109019/NVC[3]	A*02	A*32	B*07	B*57	Cw*06	Cw*07	Bw6	Bw4
109017/NVC	A*03	A*24	B*08	B*52	Cw*05	Cw*07	Bw6	Bw4
106006/NVC	A*02	A*32	B*14	B*81	Cw*02	Cw*18	Bw6	Bw6
102007/NVC	A*03	A*11	B*35	B*57	Cw*04	Cw*08	Bw6	Bw4
109018/NVC	A*01	A*11	B*07	B*08	Cw*07	Cw*07	Bw6	Bw6
110011/NVC	N/A	N/A	N/A	N/A	N/A	N/A	N/A	N/A
208003/NVC	A*23	A*23	B*41	B*57	C*07	C*07	Bw6	Bw4

[1] EC: Elite Controller
[2] VC: Virologic Controller
[3] NVC: Non-Virologic Controller

Supplementary Table S3: Cytokine production by CD4 T-cells stimulated through TCR in the presence of either IL-32α or IL-32γ.

Subject ID	Type of Stimulation	IL-2 pg/ml	TNFa pg/ml	IFNg pg/ml	IL-6 pg/ml	IL-17A pg/ml	IL-17F pg/ml	IL-22 pg/ml	IL-4 pg/ml	IL-5 pg/ml	IL-9 pg/ml	IL-10 pg/ml	IL-13 pg/ml
HIV^{neg} #1	TCR	4878.29	4592.92	1934.07	710.83	<569.41	508.6	3831.07	1068.66	4296.54	5046.77	2556.79	4139.03
	TCR+ IL-32α	3854.8	5434.04	2823.05	996.93	846.41	773.33	4490.04	1094.2	5684.72	6425.46	3790.26	5144.94
	TCR+ IL-32γ	6235.98	5170.65	3582.55	1802.42	1000.91	2474.81	5076.62	1006.84	4600.15	5824.1	2443.49	4684.2
HIV^{neg} #2	TCR	2283.52	563.53	672.23	438.04	<569.41	576.38	1532.03	388.93	1376.18	804.12	216.9	1279.02
	TCR+ IL-32α	3112.46	884.79	1041.68	649.35	754.87	1119.18	1925.51	408.94	1544.14	1445.29	275.1	1457.61
	TCR+ IL-32γ	3408.38	898.43	1418.17	1355.83	1149.61	1730.62	2932.07	404.99	1568.64	1490.44	236.91	1608.04
HIV^{neg} #3	TCR	2005.86	472.12	506.6	301.37	704.01	473.2	1554.49	249.8	3076.86	508.24	157.09	2141.44
	TCR+ IL-32α	2330.26	637.17	711.11	376.2	860.57	641.78	1268.21	246.39	3490.37	674.32	161.56	2247.83
	TCR+ IL-32γ	2611.22	645.96	801.24	761.45	1035.14	1375.15	2224.33	286.1	3483.6	806.34	161.56	2316.09
HIV^{+} #1	TCR	1814.68	582.53	554.44	164.99	704.01	259.29	986.46	207.43	1005.52	146.5	166.01	999.24
	TCR+ IL-32α	2373.7	995.55	783.94	196.83	770.99	337.81	1236.66	230.77	1408.12	164.29	110.42	1426.8
	TCR+ IL-32γ	2741.51	799.01	801.24	447.9	817.2	776.74	1444.42	234.28	1182.95	192.61	152.58	1190.91
HIV^{+} #2	TCR	1515.16	1265.27	993.96	421.34	927.59	866.29	2667.33	1638.2	6241.52	3851.28	1033.09	3957.84
	TCR+ IL-32α	1557.44	1650.76	1166.84	467.27	965.11	746.89	2631.34	2036.45	8063.61	5232.29	1424.91	4732.34
	TCR+ IL-32γ	1865.26	1427.06	1229.78	776.95	1436.81	2312.7	3652.4	1751.31	7025	4437.37	1060.12	4211.13
HIV^{+} #3	TCR	2095.85	493.36	<363.07	343.39	<569.41	577.76	1954.67	342.57	1783.52	1028.4	348.6	1680.74
	TCR+ IL-32α	2602.82	768.73	588.38	424.71	1012.48	1142.47	2270.4	386.22	2076.8	1363.61	531.5	1859.34
	TCR+ IL-32γ	3191.91	1038.11	1229.78	1357.01	1643.82	2299.49	3601.1	407.63	2274.52	2235.54	464.29	2267.73
